# Post-polymerization modification enabling library synthesis of highly isotactic polyacrylamides carrying different pendant groups

**DOI:** 10.1038/s42004-025-01663-3

**Published:** 2025-08-26

**Authors:** Yuehang Pan, Makoto Ouchi

**Affiliations:** Department of Polymer Chemistry, Graduate School of Engineering, Kyoto University, Kyoto, Japan

**Keywords:** Polymer synthesis, Polymerization mechanisms

## Abstract

The tacticity of vinyl polymers, i.e., the regularity of the side-chain stereochemistry, plays a crucial role in determining their physical properties. For example, the crystalline properties of isotactic polypropylene endow it with outstanding mechanical properties. In general, stereo-regulation during polymerization is greatly affected by even slight differences in the steric demand and/or position of the polar groups of the monomer side chains. In other words, no universal strategy to precisely control the tacticity for a given monomer with different pendant groups has been developed so far. Here, we provide a ground-breaking method for a library synthesis of highly isotactic polyacrylamides (99% *meso* dyad) with various pendant groups, including polar structures and di-substituted motifs. For that purpose, we designed an acrylamide monomer with a pendant that is sufficiently bulky to control the polymer tacticity that can be replaced by another pendant after polymerization. The transformable bulky monomer underwent iso-specific radical polymerization and subsequent aminolysis with a primary or secondary amine afforded a series of isotactic polyacrylamides derived from the added amine. Moreover, we clarified the isotacticity-dependent physical properties of the resulting polymers, such as their glass-transition temperature, crystalline properties, and solubility/thermo-responsibility in water, through a comparison with the corresponding atactic polyacrylamides.

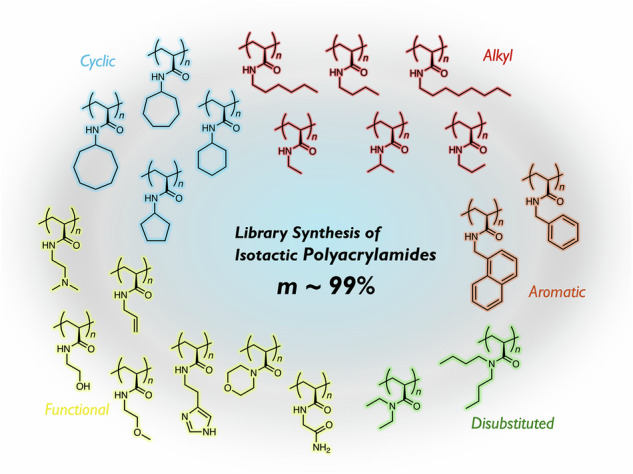

## Abstract (web summary)

The tacticity of vinyl polymers significantly influences their physical properties, yet precise control remains elusive for monomers with diverse pendant groups. Here, the authors introduce a method for synthesizing highly isotactic polyacrylamides with various pendant groups via the radical polymerization of an acrylamide monomer with a bulky pendant followed by an aminolysis transformation, revealing isotacticity-dependent physical properties.

## Introduction

**Fig. 1:**
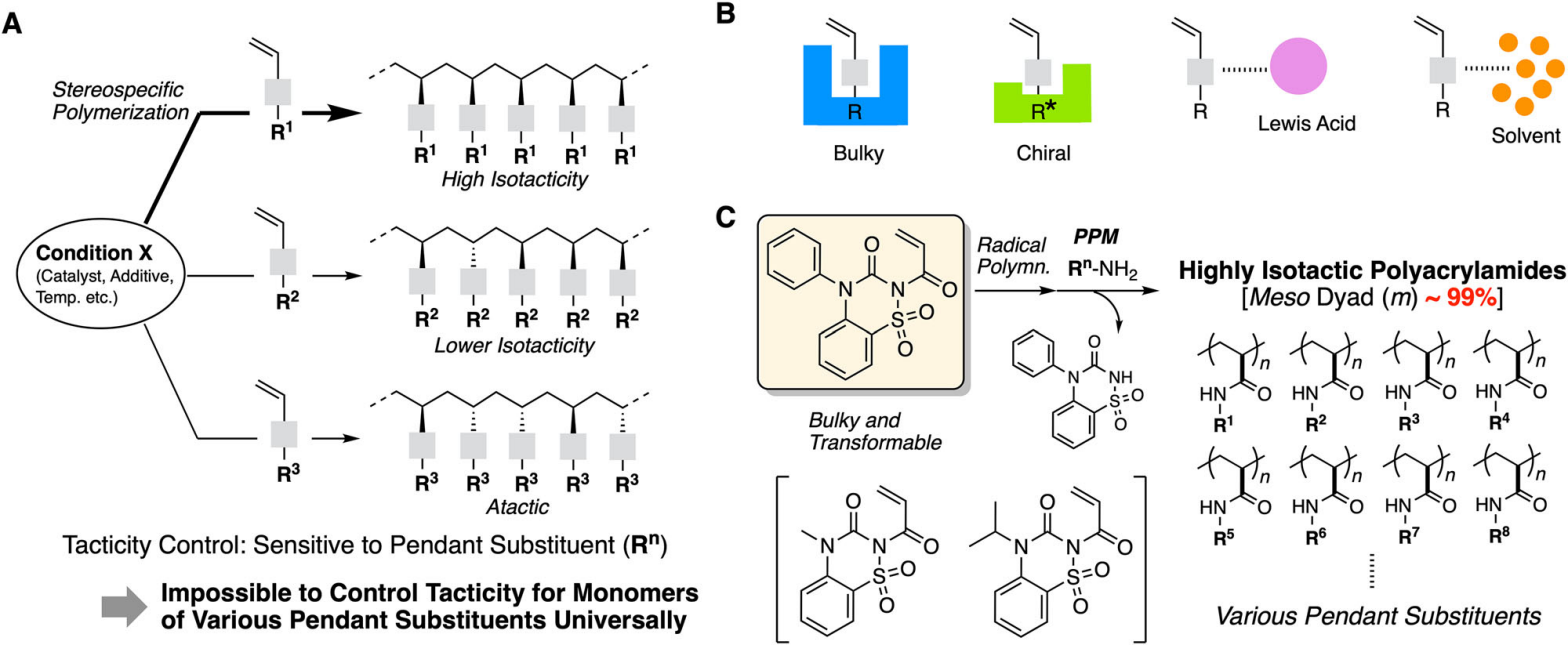
Background of tacticity control in polymerization and this work. **A** No universal method of tacticity control for various pendant groups in polymerization of a type of monomer. **B** Approaches for controlling tacticity in radical polymerization. **C** This work: design of phenyl-substituted 1,2,4-benzothiadiazine-based acrylamide monomer allowing a library synthesis of highly isotactic (*m* ~ 99%) acrylamides carrying various pendant groups (R^n^).

So far, stereoregular polymers with a high degree of tacticity have mainly been synthesized through metal-coordination polymerization and anionic polymerization^1–4^. In these methods, stereoregular propagation is highly sensitive to the steric demand and/or polarity of the substituents on the pendant groups of the monomer^5,6^, and thus, no universal strategy to precisely control the tacticity for a given monomer with different pendant groups has been developed (Fig. 1A). In particular, the stereospecific polymerization of monomers with polar functional pendant groups is highly challenging, as these polar groups can potentially thwart high levels of regular tacticity through interactions with the growing species and/or the metal catalyst^7^.

Radical polymerization has the advantages of being applicable to a wide variety of monomer derivatives and being robust against polar groups and polymerization conditions. Unfortunately, the radical process is not generally suitable for tacticity control due to the fact that the growing species is free from counterion(s) and metal coordination. To date, few approaches have been developed to control tacticity during radical polymerization based on the steric demand of the pendant group and additives that interact with pendant group^8–16^. For example, several bulky methacrylate^9^, (meth)acrylamide^10,11^ and acrylamide with chiral pendant group^12^ are known to afford stereoregular polymers. The combination of an additive such as a Lewis acid^13,14^ and hydrogen bonding compound^15,16^ or using fluorinated alcohol^17^ enables tacticity control during the radical polymerization of (meth)acrylates, vinyl acetates, and acrylamides through interaction with the pendant ester or amide groups (Fig. 1B). Efforts have also been directed toward stereo-regulation in reversible deactivation radical polymerization (RDRP) in order to achieve simultaneous control of the molecular weight and tacticity, as well as for the synthesis of stereo block copolymers^18–21^. For example, Zhong et al. have recently reported a Lewis-basic ligand on a cobalt–porphyrin radical capping agent for RDRP, in which a Lewis acid is capable of interacting with the pendant group that is present at the growing terminal. A variety of acrylamide derivatives can be applied as monomers, and the *meso* dyad content of the resulting polymers reached up to 93%^21^. However, using monomers with polar pendants resulted in a relatively low *meso*-dyad percentage, and thus, this control strategy is not universal.

Post-polymerization modification (PPM) of ester-activated acrylate or amide-activated acrylamide monomers is a useful strategy to synthesize a series of polyacrylates and polyacrylamides with identical primary structures (i.e., molecular weight, terminal groups, tacticity, and sequence) but different pendant groups^22–30^. Therefore, achieving the stereospecific polymerization of a monomer capable of undergoing PPM would enable the library synthesis of stereoregular polymers with a variety of pendant groups. Indeed, our group has recently discovered that a bulky amide-activated acrylamide monomer with an isopropyl-substituted 1,2,4-benzothiadiazine (BTD) structure (***i*Pr**-BTD) exhibits iso-specific propagation during radical polymerization; subsequent one-pot aminolysis or methanolysis reactions afforded polyacrylamides and poly(methyl acrylate)s with high levels of isotacticity^27^. The *meso*-dyad (*m*) content reached 95% when the polymerization was carried out at low temperature (–40 °C). This achievement is significant in terms of identifying novel properties and functions derived from isotacticity.

The literature on isotactic propylene indicates that when its isotacticity approaches 100%, its physical properties (e.g., crystallinity) are drastically improved^31^. The improvement in isotacticity from 95 to 100% is significant in terms of studying the physical properties. Here, we used the steric demand of the substituent on the nitrogen atom of the BTD pendant to control the tacticity. We then introduced smaller/larger substituents, i.e., methyl (**Me**-BTD) and phenyl (**Ph**-BTD) groups, than the isopropyl group used in our previous study to investigate the substituent effects on tacticity, and achieved almost 100% degree of isotacticity (*m* > 99%, Fig. 1C). Finally, we carried out a library synthesis of highly isotactic polyacrylamides with various pendant groups. We also explored the physical properties of these polymers, such as their glass-transition temperature, crystallinity, and solubility/thermal response behavior in water, and compared these properties to those of their corresponding atactic counterparts.

## Results and discussion

**Fig. 2:**
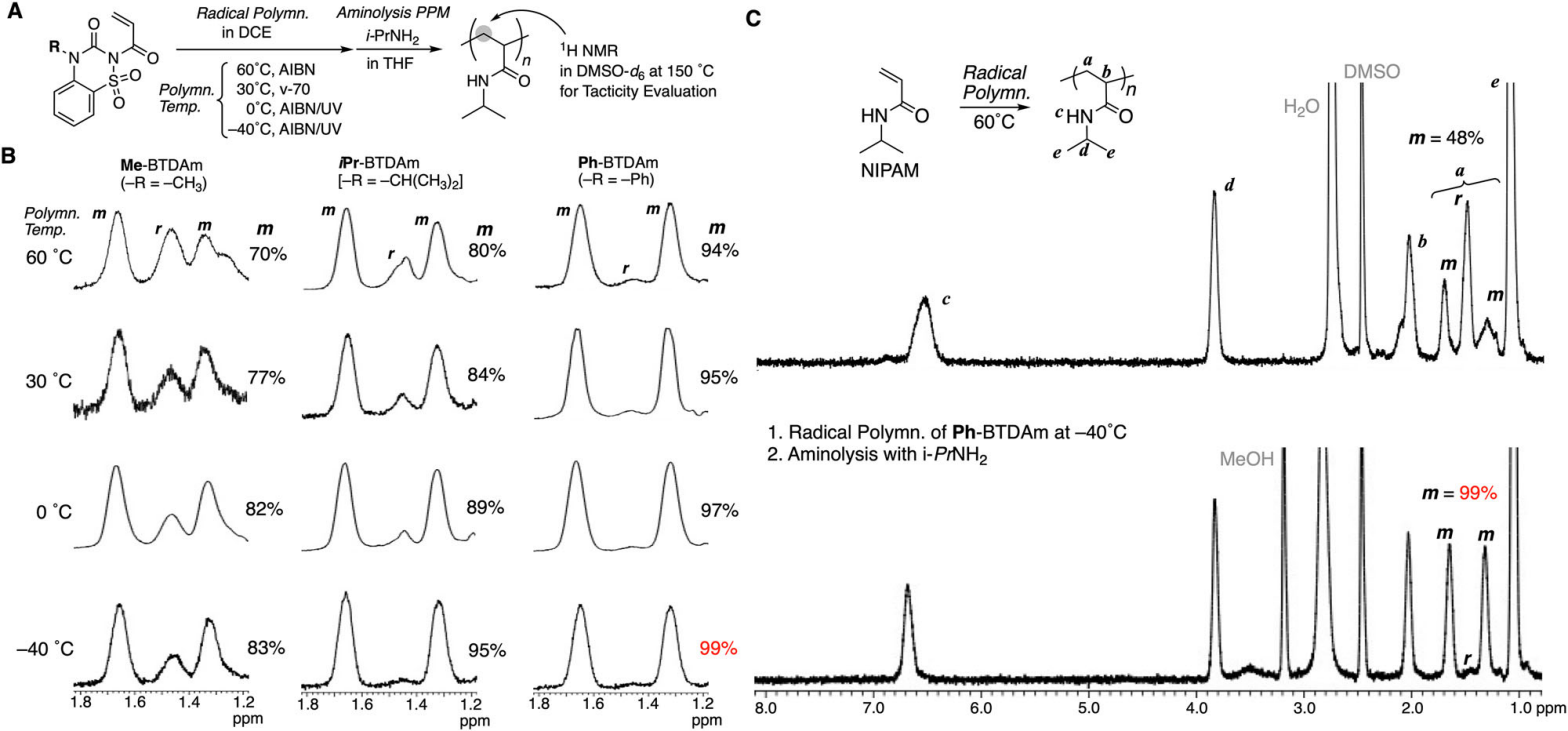
Radical polymerization of BTDAm monomers at different temperatures and post-polymerization aminolysis with *i*PrNH_2_ for the evaluation of the isotacticity of the resultant polyNIPAMs. **A** Scheme; **B** Peaks of ^1^H NMR spectra at 1.2–1.8 ppm for evaluation of *meso* dyad ratio (*m*) of polyNIPAMs; **C** ^1^H NMR spectrum of thus-obtained isotactic polyNIPAM (*m* = 99%) in comparison with that of atactic polyNIPAM (*m* = 48%) obtained via direct polymerization of NIPAM.

In our previous study with ***i*Pr**-BTDAm, we started with an evaluation of the tacticity of poly (methyl acrylate) (PMA) via a quantitative alcoholysis with methanol after the polymerization^27^. At that time, we found the addition of lithium triflate is necessary for a quantitative alcoholysis through the study on the methanolysis of the model compound for the repeating unit clarified. Therefore, we thus carried out the polymerization in the presence of lithium triflate and subsequently added methanol. The methodology of polymerization in the presence of lithium salt was adopted even for aminolysis transformation to obtain poly(alkyl acrylamide), although the lithium addition is essentially not required for the aminolysis transformation. Here, we carried out the radical polymerizations of the newly designed monomers **Me**-BTDAm and **Ph**-BTDAm as well as of ***i*Pr**-BTDAm in the absence of lithium triflate followed by aminolysis focusing on polyacrylamide for the library synthesis of the stereoregular polymer. The radical polymerizations of the three BTDAm monomers were performed at 60, 30, 0, and –40 °C with a ratio of [BTDAm monomer]_0_/[initiator]_0_ = 100/2 mM in 1,2-dichloroethane (DCE) (Fig. 2A). AIBN or v-70 were used as the initiator, and UV irradiation was applied for polymerizations at lower temperature. While the monomers were soluble in DCE, the initially transparent homogeneous polymerization solutions became heterogeneous after the polymerization was started by heating and/or UV irradiation, presumably due to the (partial) precipitation of the resulting polymers. After 4–24 h, DCE was removed under reduced pressure and replaced with tetrahydrofuran (THF) to promote the subsequent aminolysis, even though the polymers were not fully soluble in THF. Subsequently, isopropylamine (10 equiv relative to the injected amount of BTDAm monomer) was added, followed by heating to 60 °C for 24 h in order to achieve the transformation into poly(*N*-isopropylacrylamide) (polyNIPAM). In most cases, the heterogeneous solutions became partially solubilized during heating, except for the solution of ***i*Pr**-BTDAm at –40 °C and those of **Ph**-BTDAm (all temperatures), which remained insoluble, suggesting that these conditions produced polyNIPAM with higher levels of isotacticity. Depending on their solubility, the products were purified using different methods (for details, see the Supplementary Information).

The structure and tacticity of the resulting twelve polymers (i.e., from the three monomers at four different temperatures) were analyzed using ^1^H NMR spectroscopy (DMSO-*d*_*6*_, 150 °C; Fig. 2B). The spectra of all the polymers clearly exhibited peaks derived from polyNIPAM (Figs. S1–3, see the Supplementary Information). The sharpness of the peaks, which is affected by the tacticity, depends on the substituent on the monomer and the polymerization temperature. The tacticity was evaluated based on the peaks of the backbone methylene protons at 1.2–1.8 ppm, and the dyad tacticity [*meso* (*m*) and *racemo* (*r*)] was determined based on the integral ratio. In the spectra of all the products, the peaks derived from the *meso* configuration were dominant, indicating high levels of isotacticity. The following trends were observed: the *m* content increased with decreasing reaction temperature, i.e., *m* decreased in the order **Me**-BTDAm < ***i*Pr**-BTDAm < **Ph**-BTDAm. It is noteworthy that **Ph**-BTDAm gave polyNIPAM with a high *m* value (94%) even when the polymerization was performed at high temperature (60 °C). When the polymerization of **Ph**-BTDAm was carried out at lower temperature (–40 °C), the ^1^H NMR spectrum of the resultant polyNIPAM was much sharper than that of polyNIPAM prepared via the typical radical polymerization of NIPAM (Fig. 2C). For the former, the peak corresponding to the *racemo* configuration was hardly observable, and the *m* value was calculated to be > 99%. The clear difference in the isotacticity depending on the substituent of the starting monomer indicates that the steric demand of the BTDAm monomer greatly affects the stereo-selectivity during propagation. This steric-demand-dependent isotacticity is presumably related to the radical polymerization of bulky methacrylates via the formation of the gauche-staggered conformation leading to a helix^8^. In the case of methacrylates, the α-methyl group is essential for the formation of a helix during iso-specific propagation. On the other hand, for the BTDAm motif, the two bulky substituents on the nitrogen of the amide group close to backbone are likely responsible for the helical conformation leading to isotactic regulation. The result stands in sharp contrast to bulky acrylate monomers, which are known to provide atactic or slightly syndiotactic polymers^8^.

**Fig. 3:**
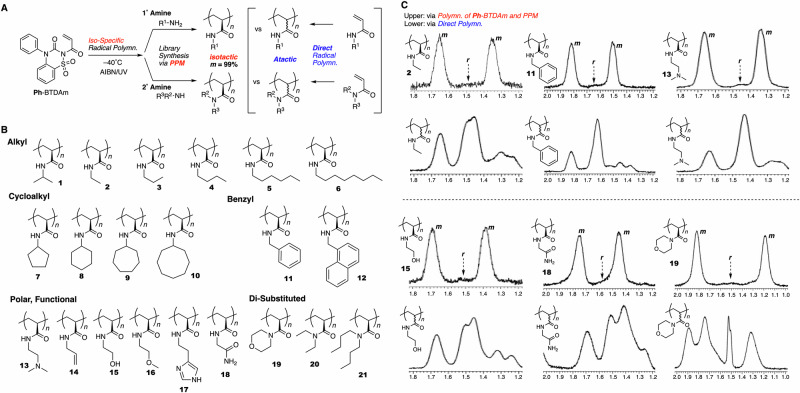
Library synthesis of isotactic polyacrylamides (*m* = 99%) with various pendant groups via the radical polymerization of Ph-BTDAm at −40 °C and subsequent aminolysis with various amines. **A** Scheme. **B** The 21 isotactic polyacrylamides synthesized in this work. **C** ^1^H NMR (1.0–2.0 ppm) spectra of selected products (**2,** **11,** **13,** **15,** **18**, and **19**, upper), together with those of the corresponding atactic polyacrylamides (lower), demonstrating the clear differences in peak shape.

The finding that the radical polymerization of **Ph**-BTDAm followed by aminolysis with isopropylamine afforded highly isotactic polyNIPAM with 99% *m* encouraged us to explore the use of other primary and secondary amine derivatives at the aminolysis stage for the library synthesis of isotactic polyacrylamides (Fig. 3A). Consequently, 21 pendant groups, including linear alkyl (**1**–**6**), cyclic (**7**–**10**), benzyl (**11,** **12**), polar functional (**13**–**19**), and di-substituted (**19**–**21**) groups, were successfully incorporated onto the isotactic polyacrylamide (Fig. 3B). The conditions of the aminolysis reaction (temperature, solvent, and reaction time) and purification method after the aminolysis reaction were varied based on the pendant group. The ^1^H NMR spectra of the products (in CD_3_OD at rt, (CD_2_Cl)_2_ at 130 °C, or DMSO-*d*_6_ at 150 °C, Figs. S22–42, see the Supplementary Information) suggested a quantitative transformation to the corresponding polyacrylamides. Unfortunately, for some polyacrylamides, the *m* value could not be estimated accurately due to overlap of the backbone methylene proton peaks used for tacticity evaluation with those of the pendant protons. However, all polymers clearly gave much sharper peaks than the corresponding atactic polymers, indicating highly controlled tacticity. Figure 3C compares the methylene proton of the ^1^H NMR spectra of selected products obtained via aminolysis (**2,** **11,** **13,** **15,** **18**, and **19**) with those of the corresponding atactic polyacrylamides obtained via direct radical polymerization. The aminolysis-derived products exhibited two main peaks with an extremely small *racemo* dyad peak in between, similar to the spectrum of the isotactic polyNIPAM. Given the quantitative transformation, we concluded that all the products were isotactic polyacrylamides with an *m* of 99%. In contrast, in the Lewis-acid-addition or fluoroalcohol strategy for acrylic and acrylamide monomers, control of the tacticity of polymers with long alkyl, cyclic alkyl, or polar groups is not straightforward due to the inhibition of interaction with the carbonyl group and the limitations in terms of appropriate solvents. Notably, our strategy enabled establishing high levels of control even for these traditionally challenging polymers. The synthesis of polyacrylamides with allyl chains cannot be achieved via the direct radical polymerization of *N*-allylacrylamide due to chain transfer to the allyl group^32^; the isotatic allyl-pendant polymer (**14**) obtained here is anticipated to allow the introduction of various functional groups via thiol–ene reactions in the isotactic conformation. The successful synthesis of isotactic polyacrylamides with di-substituted amide pendants (**19**–**21**) via transformation with secondary amines is also an important achievement worthy of special mention. Measurement of the molecular weights of most of the polymers before and after the aminolysis reactions using GPC was difficult due to their relatively low solubility in solvents such as THF, DMF, and water, resulting from their high levels of isotacticity. However, benzyl-amine derivative **11** was soluble in THF despite its high isotacticity, as confirmed by ^1^H NMR spectroscopy (Fig. S32, see the Supplementary Information). Its molecular weight and dispersity were determined to be *M*_n_ = 61200 and *Ð* = 1.79, respectively.

**Fig. 4:**
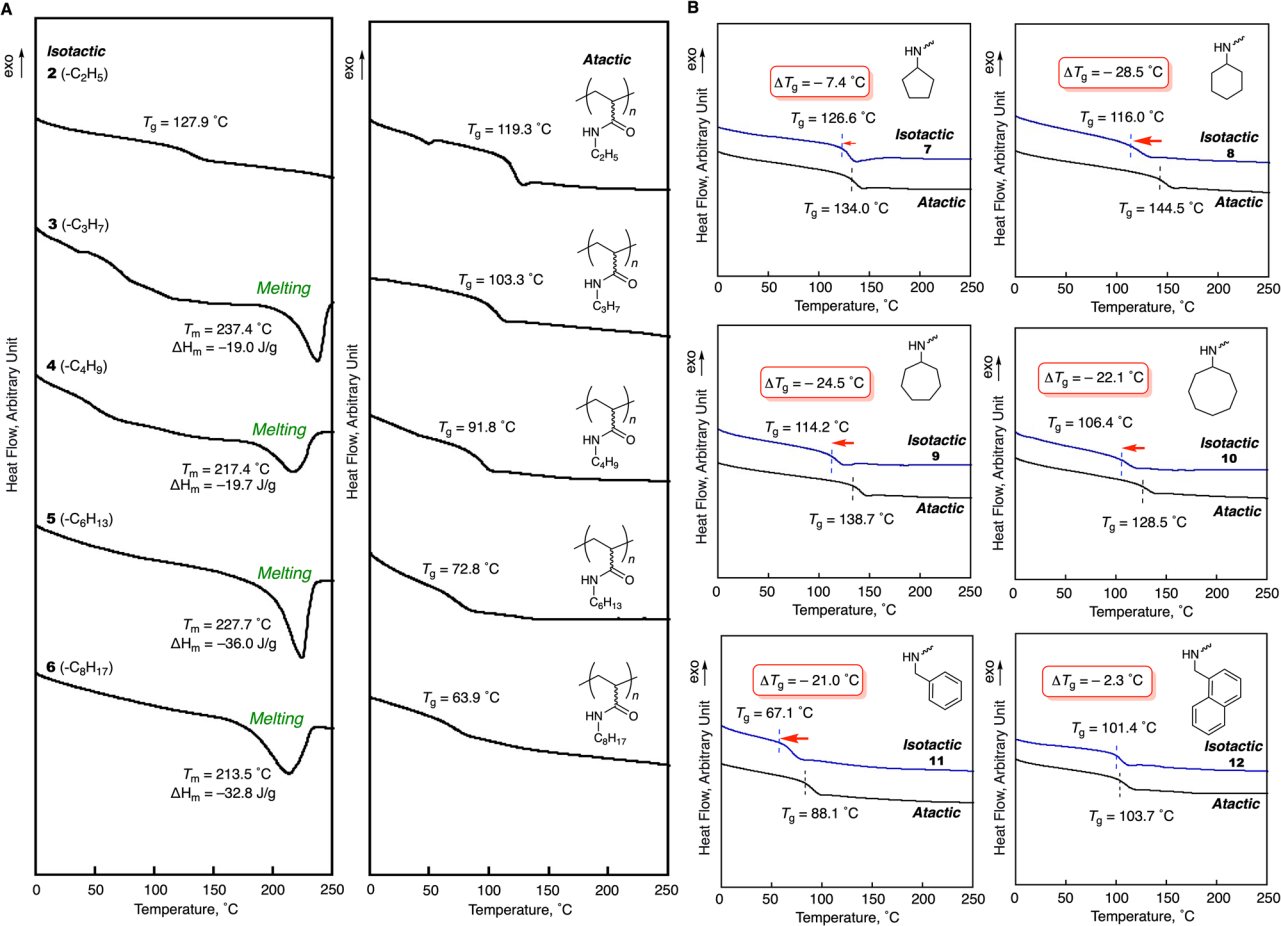
DSC thermograms (2nd heating at 10 °C/min) for evaluation of thermal property of the isotactic polyacrylamides in comparison with the atactic counterparts. **A** Evaluation of crystallization properties of isotactic polyacrylamides with linear side chains (**2**–**6**) in comparison with those of their atactic counterparts. **B** Evaluation of glass transition properties of isotactic polyacrylamides with cyclic or benzyl side chains (**7**–**12**) in comparison with those of their atactic counterparts.

Polyacrylamides or polyacrylates with long linear alkyl pendants are known to crystallize due to alkyl–alkyl interactions^33–35^. The threshold carbon number of the linear alkyl chain for polyacrylamide crystallization is twelve. The melting points (*T*_m_) of some representative polymers determined using DSC are *T*_m_ = –31 °C (–C_12_H_25_); –2.9 °C (–C_14_H_29_); 20 °C (–C_16_H_32_); 32 °C (–C_18_H_37_)^34^. These polyacrylamides are atactic because they are synthesized under typical radical polymerization conditions. The crystallinity of the atactic polyacrylamides with long alkyl chains mainly derives from the interaction between alkyl chains. On the other hand, Rajamohanan and Sanjayan have reported an isotactic *N*-acrylamide oligomer with sheet-like crystalline structures based on the intramolecular hydrogen-bonding interactions of the amide moieties^36^. We were thus interested in the crystalline features of the isotactic polyacryamides with shorter linear alkyl chains obtained in this work. We performed DSC measurements of the isotactic polyacrylamides with ethyl (–C_2_H_5,_ **2**), *n*-propyl (–C_3_H_7,_ **3**), *n*-butyl (–C_4_H_9,_ **4**), *n*-hexyl (–C_6_H_13,_ **5**), and *n*-octyl (–C_8_H_17,_ **6**) pendants and compared the resulting cooling/heating profiles with those of the corresponding atactic polymers. Figure  4A shows the DSC profiles during the second heating process after the first heating and cooling process. Polymers **3**–**6** exhibited exothermic peaks during the first cooling profile (Fig. S5, see the Supplementary Information), i.e., the endothermic peaks are likely due to a melting of the crystallized structures. On the other hand, short-alkyl-pendant polymer **2** (ethyl) did not show peaks derived from crystallization and melting. The melting temperatures (*T*_m_s) of the crystallized polymers were >200 °C [*T*_m_ = 237.4 °C (–C_3_H_7,_ **3**); 217.4 °C (–C_4_H_9,_ **4**); 227.7 °C (–C_5_H_11,_ **5**); 213.5 °C (–C_6_H_13,_ **6**)], which are much higher than those of the atactic polyacrylamides with long alkyl chains, indicating the formation of a completely different type of crystallinity. The crystalline formation was confirmed by X-ray diffraction (XRD), where relatively sharp signals were observed at around 20.0° compared to atactic polymers (Fig. S6, see the Supplementary Information). The crystalline structure is likely based on intra-sidechain or inter-sidechain interactions between amide groups in the isotactic conformation, and that a linear alkyl group with three or more carbons is essential to the crystallinity. The corresponding atactic polymers did not give such peaks derived from crystallization and melting and only base-line shifts from glass-transition temperatures (*T*_g_s) were observed.

The regularity of the pendant direction or tacticity can affect the chain conformation as well as the balance between intra-chain and inter-chain interactions between pendant groups^37^. Therefore, some vinyl polymers show tacticity-dependent glass-transition temperatures (*T*_g_). For instance, isotactic poly(methyl methacrylate) is known to show a lower *T*_g_ than its atactic and syndiotactic counterparts due to the higher free volume caused by the helical conformation^38^. On the other hand, the *T*_g_ of polystyrene is barely affected by its tacticity^39^. In the case of *N*-substituted polyacrylamides, the tacticity can have a significant impact on *T*_g_ through its influence on the nature of the hydrogen-bonding interactions between amide groups (intramolecular vs intermolecular). In fact, the tacticity dependence of the glass-transition temperature of polyNIPAM has previously been studied from the viewpoint of the hydrogen-bonding interactions of the amide group^40^. We thus measured the *T*_g_ values of the obtained isotactic polyacrylamides with cyclic groups (**7**–**10**) and benzyl derivatives (**11** and **12**), which did not exhibit crystallization peaks in the DSC measurements and compared these with those of the corresponding atactic polymers (Fig. 4B). Most of the isotactic polyacrylamides (**8**–**11**) showed *T*_g_ values of more than 20 °C lower than those of their atactic counterparts. These polymers likely formed helical conformations stabilized by the hydrogen-bonding interactions of the amide groups due to the isotactic regulation. On the other hand, the *T*_g_ of the isotactic polyacrylamide with a naphthyl group (**12**) was almost the same as that of its atactic counterpart. This substituent may disfavor the adoption of the helical conformation even in the isotactic conformation due to the π–π stacking interactions of the naphthalene moieties predominating over the amide hydrogen-bond interactions.

Polyacrylamides with short alkyl chains or polar groups are soluble in water, and some exhibit thermally responsive solubility^41^. PolyNIPAM and poly-*N*,*N*-diethylacrylamide (polyDEAm) are representative polymers that show a lower critical solution temperature (LCST)^42^ in water, i.e., their solutions are transparent below the LCST, but become turbid above the LCST. A plausible mechanism for the thermal response behavior of polyNIPAM is the cooperative dehydration of water molecules from the amide groups in the multitude of repeating units, leading to a sharp phase-transition response. Therefore, the tacticity of polymers can affect their solubility behavior. Indeed, it has been reported that the cloud point (*T*_cp_) of polyNIPAM decreases with increasing isotacticity, and that the highly isotactic polyNIPAM is insoluble in water^43,44^. On the other hand, Zhong et al. have recently reported that polyDEAm exhibits the opposite trend, i.e., the *T*_cp_ increases with increasing isotacticity^21^.

**Fig. 5:**
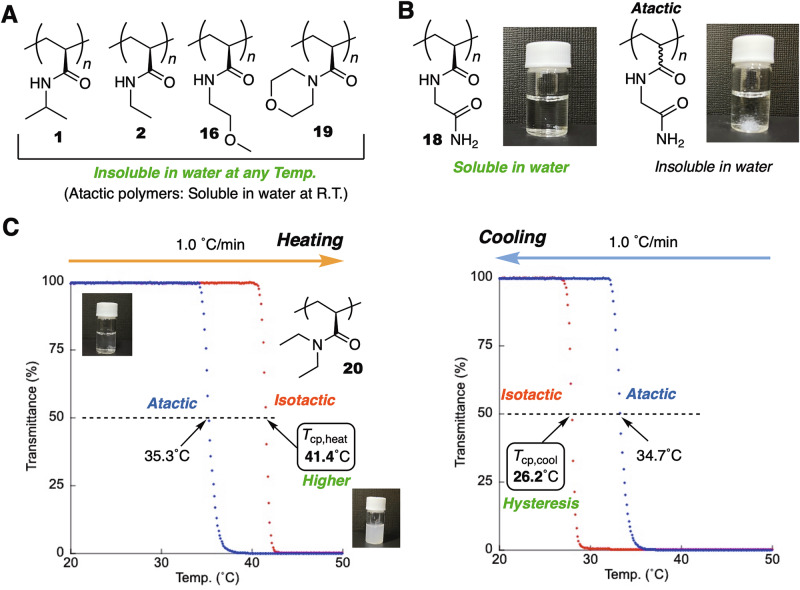
Solubilities of isotactic polyacrylamides in water. **A** Isotactic polyacrylamides that are insoluble in water (**1,** **2,** **16**, and **19**) and the corresponding atactic polymers, which are soluble in water. **B** An isotactic polyacrylamide that is soluble in water (**18**) together with the corresponding atactic polymer, which is insoluble in water. **C** Variable-temperature UV−vis transmittance measurements (*λ* = 670 nm, heating/cooling rate = 1.0 °C/min) of an aqueous solution of **20** (0.25 wt%), together with those of the corresponding atactic polymer.

We thus subsequently investigated the water solubility of the isotactic polyacrylamides with short alkyl chains or polar groups. Isotactic polyNIPAM (**1**) with an *m* of 99% was not soluble in water, and its solubility in water did not show thermal responsiveness; similar behavior has been reported for polyNIPAM (*m* = 95–96%) in the literature (Fig. 5A)^27,44^. The isotactic polyacrylamides with ethyl (**2**), 2-methoxyethyl (**16**), and morpholine (**19**) pendants were also insoluble in water at any temperature. This result stands in sharp contrast to the behavior of the corresponding atactic polymers, which are highly soluble in water regardless of temperature. Poly(*N*-acryloyl glycinamide) (PAcGAm) is known to exhibit a sharp upper critical solution temperature in water, i.e., the atactic polymer is insoluble in water at ambient temperature due to hydrogen-bond interactions between chains, albeit that these interactions are broken at higher temperature, leading to solubilization (Fig. 5B)^45^. Conversely, isotactic polymer **18** was soluble in water even at room temperature, and no thermal response was observed. This polymer probably features intra-chain rather than inter-chain hydrogen-bond interactions in the isotactic conformation, thus making the isotactic polymer more soluble in water.

The isotactic polyacrylamide with two ethyl substituents (**20**) was soluble in water, albeit that the solution became turbid upon heating (Fig. 5C). We measured the variable-temperature transmittance of the water solution (2.5 mg/mL) using UV-vis spectroscopy (*λ* = 670 nm, 10 °C/min) to characterize the thermal response and cloud point (*T*_cp_; temperature at 50% transmittance). The transmittance of the solution decreased rapidly at ~41 °C during the heating process, and the *T*_cp_ during the heating process (*T*_cp,heat_) was observed at 41.5 °C, which was higher than that of its atactic counterpart polyDEAm (*T*_cp,heat_ = 35.2 °C). Interestingly, the isotactic polymer exhibited a much greater hysteresis. The *T*_cp_ of **20** during the cooling process (*T*_cp,cool_) was much lower (*T*_cp,cool_ = 27.9 °C) than its *T*_cp, heat_ value; in contrast, atactic polyDEAA exhibited very similar *T*_cp,cool_ (33.2 °C) and *T*_cp,heat_ (35.2 °C) values. Presumably, the isotactic polyDEAm forms tighter aggregates upon heating due to its high stereoregularity and thus is more difficult to hydrate, leading to the unusual hysteresis behavior.

The library synthesis of highly isotactic polyacrylamides via the PPM strategy with **Ph**-BTDAm and the novel physical properties derived from their isotacticity could have a significant impact on polymer and materials science. However, the leaving group from the **Ph**-BTDAm unit, i.e., phenyl 1,2,4-benzothiadiazine (**Ph**-BTD), is discarded during the PPM process, which is not favorable in terms of atom economy. We thus subsequently examined the possibility of recovering and reusing the leaving group (Fig. S7, see the Supplementary Information). As a model experiment, we conducted the radical polymerization of **Ph**-BTDAm at –40 °C followed by the addition of an excess of isopropyl amine. The solution was poured into a 10-fold excess of methanol to precipitate the isotactic polyNIPAM, and **Ph**-BTD was recovered from the methanol layer. For that purpose, methanol was removed from the supernatant solution under reduced pressure, and the crude compound was dissolved in a 1:1 mixture of H_2_O and MeOH in the presence of an excess of NaHCO_3_ (saturated) to convert the acidic amide compound to its sodium salt, followed by the addition of HCl to precipitate the crystallized compound. The precipitate was then recovered via filtration. The ^1^H and ^13^C NMR spectra supported the recovery of **Ph**-BTD in 79% yield. Reaction of the recovered **Ph**-BTD with acryl chloride would yield **Ph**-BTDAm. Although further improvements are needed, this recovery process could lead to the development of a sustainable strategy to synthesize isotactic polyacrylamides.

## Conclusions

In summary, we have achieved the synthesis of highly isotactic polyacrylamides (*m* = 99%) via the radical polymerization of a monomer with a transformable pendant followed by an aminolysis transformation. The transformation proceeded quantitatively using a variety of amine compounds, leading to a library of isotactic polyacrylamides with different pendant groups. A comparison of their physical properties with those of the corresponding atactic polyacrylamides revealed tacticity-dependent behavior regarding crystallization, glass transition, and solubility/thermal response in water. The BTDAm motif is promising in terms of design extensibility, as substituents could be introduced on the benzene ring to improve the solubility toward control of RDRP as well as efficient recycling processes. Therefore, the construction of various architectures containing isotactic polyacrylamide segments, such as block copolymers, graft copolymers, and branch polymers, will be accessible, providing a path toward polymers with more advanced properties/functions.

## Methods

### Synthetic procedures

See “Synthetic Procedures” and “Procedures of Polymerization and Post-Polymerization Modification (PPM)” in Supplementary Information (Contents 2 and 3).

Physical Properties of Isotactic Polyacrylamides in Comparison with Atactic Counterparts

See DSC Curves and XRD Charts in Supplementary Information (Contents 4 and 5)

Physical Properties of Isotactic Polyacrylamides in Comparison with Atactic Counterparts

See DSC Curves and XRD Charts in Supplementary Information (Contents 4 and 5)

Recycle of Pendant Group

See “Recycle of Pendant Group” in Supplementary Information (Content 6)

NMR Spectra

See “Supplementary Data” in Supplementary Information (Content 7).

## Supplementary information

Supplemental information

## Data Availability

The data supporting the findings of this study are available within the article and its Supplementary Information. All relevant data are available from the authors upon request.
