# Supplementary material for: Post-polymerization modification enabling library synthesis of highly isotactic polyacrylamides carrying different pendant groups
Source: Commun Chem. 2025 Aug 26;8:259. doi: 10.1038/s42004-025-01663-3 (PMC12381277; doi:10.1038/s42004-025-01663-3)
Supplement: Supplementary file 1 — Supplemental information [file 42004_2025_1663_MOESM1_ESM.pdf]

Supplemental information

Post-Polymerization Modification Enabling Library Synthesis of Highly Isotactic Polyacrylamides Carrying Different Pendant Groups

Yuehang Pan and Makoto Ouchi*

Department of Polymer Chemistry, Graduate School of Engineering, Kyoto University, Nishikyo-ku, Kyoto 615-8510, Japan
ouchi.makoto.2v@kyoto-u.ac.jp

Contents

1. General Information

2. Synthetic Procedures

- **2.1. Syntheses of Monomers**
- **2.2. Syntheses of *N*-Alkyl Acrylamide Monomers for Atactic Polymers**

3. Procedures of Polymerization and Post-Polymerization Modification (PPM)

- **3.1. Polymerizations of BTDAm Monomers at Different Temperatures and Transformation into Poly(*N*-isopropylacrylamide) for Tacticity Evaluation**
- **3.2. Syntheses of Isotactic Polyacrylamides with PhBTDAm**
- **3.3. Syntheses of Atactic Polyacrylamides**

4. DSC Thermograms of Isotactic and Atactic Polyacrylamides

5. XRD of Isotactic Polyacrylamides with Linear Side Chains (vs Atactic Counterparts)

6. Recycle of Pendant Group

7. Supplementary Data

- **7.1. ^{1}H and ^{13}C NMR Spectra of BTDAm Monomers**
- **7.2. ^{1}H and ^{13}C NMR Spectra of *N*-Alkyl Acrylamide Monomers**
- **7.3. ^{1}H NMR Spectra of Isotactic/Atactic Polymers**

1. General Information

NMR spectra were recorded on a JEOL JNM-ECA500 spectrometer operating at 500.16 MHz (^{1}H NMR) and 125.04 MHz (^{13}C NMR). Number-average molecular weight (M_n), weight-average molecular weight (M_w), and molecular weight distribution ($Đ$) of polymers were measured by size exclusion chromatography (SEC) at 40℃ in THF or DMF (solution of 10 mM LiBr) as an eluent on three polystyrene-gel columns. The columns were calibrated using PMMA standards (Polymer Laboratories: M_n = 800–2200000). For SEC in THF: Shodex LF-404 (column); exclusion limit = 2 × 10^6; particle size = 6 µm; pore size = 3000 Å; 0.46 cm i.d. × 25 cm; flow rate, 0.35 mL min^{-1} connected to HLC-8320GPC (TOSOH). For SEC in DMF: Shodex KF-805L (column); exclusion limit = 4 × 10^6; particle size = 10 µm; pore size = 5000 Å; 0.8 cm i.d. × 30 cm; flow rate, 1.0 mL min^{-1} connected to a PU-2080 precision pump, a RI- 2031 refractive-index detector, and a UV-2075 UV/vis detector (all from JASCO). Thermoresponse of polymers in water was evaluated by temperature variable transmittance measurement with UV-Vis (Jasco V-750 spectrophotometer, optical path length = 1.0 cm, l = 670 nm). Glass transition temperature (T_g) of polymer samples was measured by a DSC Q200 calorimeter (TA instruments) equipped with RCS 90 electric machine under dry nitrogen flow. Cast films (2-6 mg) on aluminum pan (Tzero pan) were used as samples for DSC measurement. The temperature program was as follows:

1. Heating to 260 ˚C at 10 ˚C/min from 40 ˚C (1st heating)
2. Keeping at 260 ˚C for 5 min
3. Cooling to –10 ˚C at 10 ˚C/min
4. Keeping at –10 ˚C for 5 min
5. Heating to 260 ˚C at 10 ˚C/min (2nd heating)
6. Keeping at 260 ˚C for 5 min
7. Cooling to –10 ˚C at 10 ˚C/min
8. Keeping at –10 ˚C for 5 min
9. Heating to 260 ˚C at 10 ˚C/min (3rd heating)
10. Keeping at 260 ˚C for 5 min

T_g was determined from the 2nd heating process and the adequacy or repeatability was confirmed by the 3rd heating process.

X-ray diffraction (XRD) spectra were recorded on an Aeris (Malvern) operating at 40 kV and 15 mA with Cu Kα radiation (λ = 0.154 nm). Samples were annealed at 220 ˚C for 1 h. The scanning rate of the scattering angle (2θ) was set to 0.0435°/s in the range of 5−60°.

For purification with column chromatography, Wako gel C200 (Wako) and mixture solvents of *n*-hexane (Wako, >96%) and ethyl acetate (AcOEt, Wako, >99.5%) with some volume ratios were used. For polymer purification by dialysis, MWCO1000 (Spectra/PorVR7, diameter 29 mm) was used as the membrane and methanol (Wako, >99.5%) was used as the solvent. All other materials were purchased from Wako, TCI, or Sigma Aldrich and used as received.

2. Synthetic Procedures

2.1. Syntheses of Monomers

2.1.1. Methyl-Substituted Monomer (**Me**-BTDAm)

Synthesis of the pendant (**Me**-BTD)

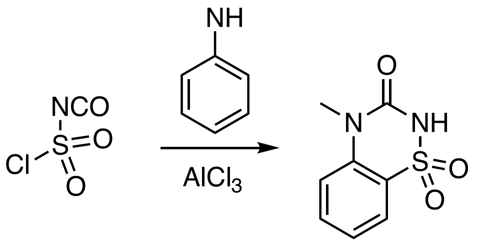

Me-BTD

The methyl-substituted pendant, (**Me**-BTD: 4-methyl-2H-benzo[e][1,2,4]thiadiazin-3(4H)-one 1,1-dioxide), was synthesized by a method slightly modified from the previously reported one[1]. A solution of *N*-methylaniline (34.0 mL, 314 mmol) in nitromethane (175.0 mL) was added to the solution of chlorosulfonyl isocyanate (30.0 mL, 1.1 equiv., 348 mmol) in nitromethane (175.0 mL) at –40 ˚C. The mixture was stirred for 15 min before adding 46.0 g of $AlCl_3$ (1.1 equiv., 345 mmol), and the mixture was kept stirring at 110 ˚C for 45 min. The resultant solution was cooled and poured onto a saturated solution of $NaHCO_3$ in H_2O/MeOH (1:1, v/v, 2000 mL). The mixture was treated with charcoal, followed by centrifugation to obtain the supernatant. The homogeneous solution was acidified by HCl to pH 1, and the precipitate was filtered in vacuo to obtain **Me**-BTD as a white solid (21.2 g, 31.9% yield).

^{1}H NMR (500 MHz, acetone-d_6): δ = 10.81 (brs, 1H), 7.86 (dd, 1H), 7.78 (m, 1H), 7.53 (d, 1H), 7.40 (m, 1H), 3.51 (s, 1H). ^{13}C NMR (125 MHz, acetone-d_6): δ 150.04, 137.69, 134.31, 126.33, 123.45, 129,96, 116.83, 31.32.

Synthesis of **Me**-BTDAm

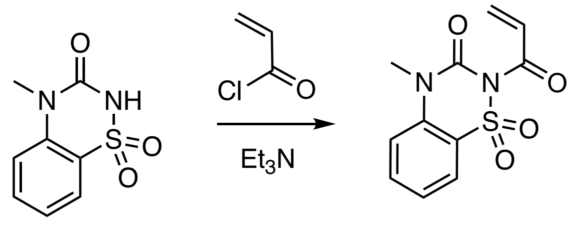

The methyl-substituted pendant (**Me**-BTD, 3.00 g, 14.1 mmol) and phenothiazine (30.0 mg, 0.150 mmol) as an inhibitor for radical polymerization was placed in round-bottom-flask and was dissolved in THF (30.0 mL). Then, 2.2 ml of triethylamine (1.10 eq, 15.8 mmol) was added and subsequently 1.2 ml of acryloyl chloride (1.05 eq, 14.9 mmol) was slowly added at 0 ˚C. The mixture was stirred for 1 h, and the solvent was evaporated from the reaction solution. The residue was dissolved in CH_2Cl_2 and the solution was washed with saturated $NaHCO_3$ aqueous solution and brine. The organic layer was dried over anhydrous Na_2SO_4 and evaporated. Note that the temperature should be kept below 30 ˚C during evaporation: otherwise the polymerization may occur. The obtained solid was washed with diethyl ether and hexane, followed by recrystallization from the solution in 1,2-dichloroethane at -40 ˚C to yield the corresponding acrylamide monomer (**Me**-BTDAm) as a light grey solid (1.7 g, yield 45.2%).

^{1}H NMR (500 MHz, $CDCl_3$): δ = 7.94 (dd, 1H), 7.74 (m, 1H), 7.37 (m, 1H), 7.33 (d, 1H), 6.54 (dd, 1H), 6.39 (dd, 1H), 5.93 (dd, 1H), 3.57 (s, 3H). ^{13}C NMR (125 MHz, $CDCl_3$): δ 163.44, 149.49, 137.07, 134.95, 132.95, 130.60, 127.12, 124.50, 123.71, 117.05, 33.31.

2.1.2. Isopropyl-Substituted Monomer (***i*Pr**-BTDAm)

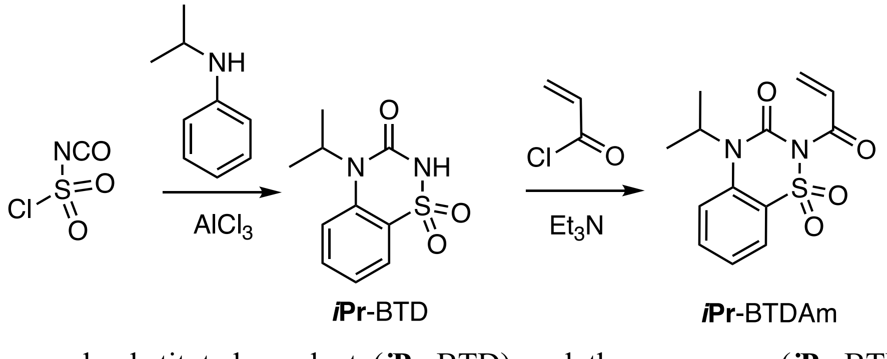

The isopropyl-substituted pendant (***i*Pr**-BTD) and the monomer (***i*Pr**-BTDAm) were synthesized by the previously reported method[2].

***i*Pr**-BTD: ^{1}H NMR (500 MHz, $CDCl_3$): δ = 7.88 (dd, 1H), 7.65 (m, 1H), 7.39 (d, 1H), 7.32 (m, 1H), 4.53 (m, 1H), 1.65 (d, 6H). ^{13}C NMR (125 MHz, $CDCl_3$): δ 150.75, 138.13, 133.84, 127.74, 123.93, 122.67, 117.83, 52.86, 20.26.

***i*Pr**-BTDAm: ^{1}H NMR (500 MHz, $CDCl_3$): δ = 7.90 (dd, 1H), 7.69 (m, 1H), 7.41 (d, 1H), 7.35 (m, 1H), 6.52 (dd, 1H), 6.38 (dd, 1H), 5.91 (dd, 1H), 4.50 (m, 1H), 1.65 (d, 6H). ^{13}C NMR(125 MHz, $CDCl_3$): δ 163.46, 148.76, 137.93, 134.52, 132.40, 130.67, 128.70, 124.62, 123.68, 118.77, 54.40, 20.22.

2.1.3. Phenyl-Substituted Monomer (**Ph**-BTDAm)

Synthesis of the pendant (**Ph**-BTD)

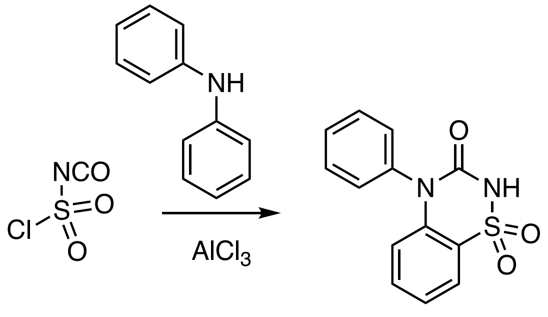

Ph-BTD

The phenyl-substituted pendant, 4-phenyl-2H-benzo[e][1,2,4]thiadiazin-3(4H)-one 1,1-dioxide (**Ph**-BTD), was synthesized by a method slightly modified from the previously reported one[1]. A solution of chlorosulfonyl isocyanate (14.0 ml, 162.2 mmol, 1.1 equiv for diphenylamine) in nitromethane (80 mL) was combined with 25.0 g of diphenylamine (147.7 mmol) in nitromethane (80 mL) at -40 ˚C. The mixture was stirred for 15 min before addition of $AlCl_3$ (19.7 g, 147.8 mmol, 1.1 equiv for diphenylamine), and the solution was heated to 110 ˚C and stirred for 45 min. The resultant solution was cooled and poured into a large volume of ice water (1.2 L). The precipitation was filtered and washed several times with cold toluene and hexane. The crude was solved in hot ethanol, followed by cooling to recrystallize the pure product as a white solid (25.3 g, yield 62.4%).

^{1}H NMR (500 MHz, $CDCl_3$): δ = 7.92 (dd, 1H), 7.55 (m, 3H), 7.45 (m, 1H), 7.37 (m, 2H), 7.30 (m, 1H), 6.63 (d, 1H). ^{13}C NMR (125 MHz, $CDCl_3$): δ 149.75, 137.97, 136.19, 133.99, 130.40, 129.68, 129.28, 125.62, 124.10, 122.54, 118.69.

Synthesis of **Ph**-BTDAm

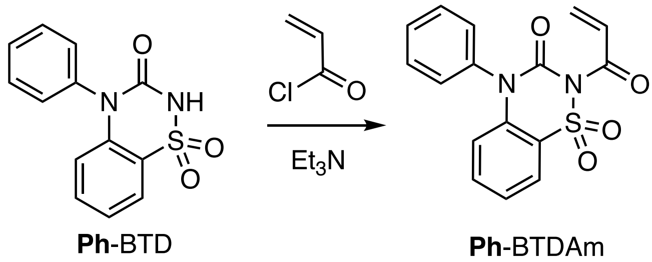

The phenyl-substituted pendant (**Ph**-BTD, 10.0 g, 36.5 mmol) and phenothiazine (100.0 mg, 0.50 mmol) as an inhibitor for radical polymerization was placed in round-bottom-flask and was dissolved in THF (100 mL) and acetone (200 ml). Triethylamine (5.6 ml, 40.2 mmol, 1.10 equiv) was then added, and acryloyl chloride (3.1 ml, 38.4 mmol, 1.05 equiv) was slowly added at 0 ˚C. The mixture was stirred for 30 min, and the solvent was evaporated from the reaction solution. The residue was dissolved in CH_2Cl_2 and the solution was washed with H_2O and brine. The organic layer was dried over anhydrous Na_2SO_4 and evaporated. The obtained solid was washed with diethyl ether and hexane, followed by recrystallization from the solution in 1,2-dichloroethane at -40 ˚C to yield **Ph**-BTDAm as a white solid (8.6 g, yield 71.8%).

^{1}H NMR (500 MHz, $CDCl_3$): δ = 7.97 (dd, 1H), 7.54 (m, 5H), 7.39 (m, 2H), 7.34 (m, 1H), 6.72 (d, 1H), 6.58 (dd, 1H), 6.48 (dd, 1H), 5.96 (dd, 1H). ^{13}C NMR (125 MHz, $CDCl_3$): δ = 163.45, 148.87, 137.64, 136.40, 134.48, 133.02, 130.59, 130.39, 129.79, 129.07, 126.98, 124.63, 123.44, 119.46.

2.2. Syntheses of *N*-Alkyl Acrylamide Monomers for Atactic Polymers

General Procedure

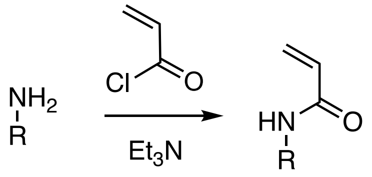

To a solution of amine (1.0 equiv, 50 mmol) in dry THF (100 mL) was added triethylamine (8.32 mL, 1.2 equiv, 60 mmol) and acryloyl chloride (4.24 mL, 1.05 equiv, 52.5 mmol) at 0 ℃ in this order. After stirring for 3 h, the reaction mixture was filtered, and the filtrate was concentrated under reduced pressure. The crude product was purified by silica column chromatography (eluted by a mixture of *n*-hexane and AcOEt) to afford *N*-acrylacrylamide compound.

N-Hexylacrylamide (HexAm)

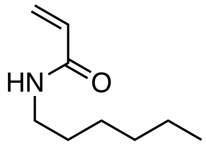

N-Hexylacrylamide was obtained as a white solid (yield: 95%) after purification by silica column chromatography (eluent: *n*-hexane/AcOEt = 2/1). ^{1}H NMR (500 MHz, $CDCl_3$): δ = 6.26 (dd, 1H), 6.12 (dd, 1H), 5.61 (dd, 1H), 3.32 (m, 2H), 1.53 (m, 2H), 1.30 (m, 6H), 0.88 (t, 3H). ^{13}C NMR (125 MHz, $CDCl_3$): δ = 165.67, 131.15, 126.07, 39.72, 31.56, 29.60, 26.70, 22.62, 14.08.

N-Octylacrylamide (OctAm)

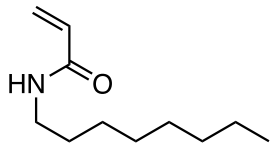

N-Octylacrylamide was obtained as a white solid (yield: 93%) after purification by silica column chromatography (eluent: *n*-hexane/AcOEt = 2/1). ^{1}H NMR (500 MHz, $CDCl_3$): δ = 6.27 (dd, 1H), 6.09 (dd, 1H), 5.62 (dd, 1H), 3.32 (m, 2H), 1.53 (m, 2H), 1.28 (m, 10H), 0.88 (t, 3H). ^{13}C NMR (125 MHz, $CDCl_3$): δ = 165.56, 131.07, 126.23, 39.73, 31.87, 29.67, 29.34, 29.28, 27.03, 22,72, 14.17.

N-Cyclopentylacrylamide (cPenAm)

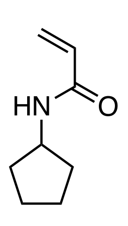

N-Cyclopentylacrylamide was obtained as a white solid (yield: 99%) after purification by silica column chromatography (eluent: *n*-hexane/AcOEt = 2/1). ^{1}H NMR (500 MHz, $CDCl_3$): δ = 6.27 (dd, 1H), 6.07 (dd, 1H), 5.62 (dd, 1H), 4,29 (m, 1H), 2.03 (m, 2H), 1.65 (m, 4H), 1.41 (m, 2H). ^{13}C NMR (125 MHz, $CDCl_3$): δ = 165.13, 131.13, 126.19, 51.31, 33.25, 23.84.

N-Cyclohexylacrylamide (cHexAm)

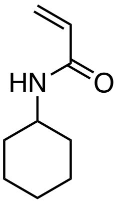

N-Cyclohexylacrylamide was obtained as a white solid (yield: 48%) after purification by silica column chromatography (eluent: n-hexane/AcOEt = 2/1). ^{1}H NMR (500 MHz, $CDCl_3$): δ = 6.26 (dd, 1H), 6.08 (dd, 1H), 5.61 (dd, 1H), 3.85 (m, 1H), 1.95 (m, 2H), 1.72 (m, 2H), 1.63 (m, 1H), 1.38 (m, 2H), 1.16 (m, 3H). ^{13}C NMR (125 MHz, $CDCl_3$): δ = 164.63, 131.38, 126.07, 48.32, 33.21, 25.61, 24.94.

N-Cycloheptylacrylamide (cHepAm)

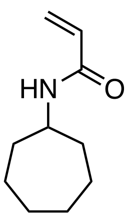

N-Cycloheptylamide was obtained as a white solid (yield: 91%) after purification by silica column chromatography (eluent: n-hexane/AcOEt = 2/1). ^{1}H NMR (500 MHz, $CDCl_3$): δ = 6.25 (dd, 1H), 6.07 (dd, 1H), 5.60 (dd, 1H), 4.03 (m, 1H), 1.96 (m, 2H), 1.62 (m, 4H), 1.96 (m, 2H), 1.48 (m, 6H). ^{13}C NMR (125 MHz, $CDCl_3$): δ = 164.37, 131.41, 126.01, 50.53, 35.17, 28.14, 24.18.

N-Cyclooctylacrylamide (cOctAm)

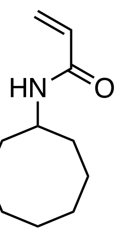

N-Cyclooctylacrylamide was obtained as a white solid (yield: 95 %) after purification by silica column chromatography (eluent: n-hexane/AcOEt = 2/1). ^{1}H NMR (500 MHz, $CDCl_3$): δ = 6.26 (dd, 1H), 6.07 (dd, 1H), 5.61 (dd, 1H), 4.08 (m, 1H), 1.86 (m, 2H), 1.70-1.49 (m, 12H). ^{13}C NMR (125 MHz, $CDCl_3$): δ = 164.34, 131.40, 126.04, 49.49, 32.30, 27.26, 25.52, 23.76.

N-1-Naphthylacrylamide (NaphAm)

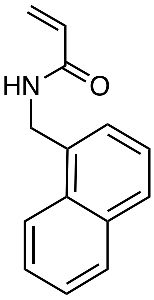

N-1-Naphthylacrylamide was obtained as a light yellow solid (yield: 37 %) after purification by silica column chromatography (eluent: n-hexane/AcOEt = 2/1). ^{1}H NMR (500 MHz, $CDCl_3$): δ = 8.02 (d, 1H), 7.88 (dd, 1H), 7.82 (d, 1H), 7.53 (m, 2H), 7.43 (m, 2H), 6.33 (dd, 1H), 6.07 (dd, 1H), 5.64 (dd, 1H), 4.96 (d, 2H). ^{13}C NMR (125 MHz, $CDCl_3$): δ = 165.20, 133.99, 133.34, 131.51, 130.61, 128.88, 127.07, 127.00, 126.85, 126.16, 125.51, 123.61, 41.98.

N-Ethylacrylamide(EtAm)

N-Ethylacrylamide was prepared by following procedure:

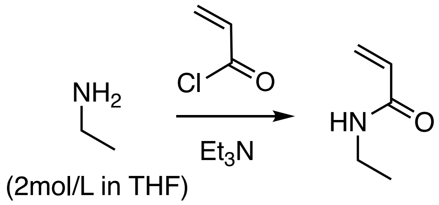

To a solution of ethylamine (1.0 equiv, 60 mmol) in THF (30 mL) was added 30 ml of dry THF, triethylamine (10.0 mL, 1.2 equiv, 71.7 mmol) and acryloyl chloride (5.1 mL, 1.05 equiv, 63.1 mmol) at 0 ℃ in this order. After 3 h stirring, the reaction mixture was filtered, and the filtrate was concentrated under reduced pressure. The resulting liquid was further purified with silica column chromatography (Hexane: AcOEt = 2:1 as eluent) to yield 5.0 g *N*-ethylacrylamide as a yellow liquid with a yield of 84%. ^{1}H NMR (500 MHz, $CDCl_3$): δ = 6.26 (dd, 1H), 6.19 (dd, 1H), 5.60 (dd, 1H), 3.36 (m, 2H), 1.17 (t, 3H). ^{13}C NMR (125 MHz, $CDCl_3$): δ = 165.82, 131.26, 125.83, 34.44, 14.70.

3. Procedures of Polymerization and Post-Polymerization Modification (PPM)

3.1. Polymerizations of BTDAm Monomers at Different Temperatures and Transformation into Poly(*N*-isopropylacrylamide) for Tacticity Evaluation

In a Schlenk tube, **Ph**-BTDAm (0.18 g, 0.55 mmol), AIBN (1.8 mg, 11.0 μmol), $(CH_2Cl)_2$ (5.3 ml), and a small portion of tetralin (internal standard) were added in this order at room temperature under dry argon to give the polymerization solution with the following concentrations: $[$**Ph**-BTDAm$]_0/[AIBN]_0$ = 100/2 mM in $(CH_2Cl)_2$. For determination of monomer conversion, a small portion of the polymerization solution was pulled out and the monomer ratio to the internal standard before polymerization was measured by ^{1}H NMR. The tube was then immersed in an oil bath at 60 ˚C to initiate polymerization. A small portion was taken from the polymerization solution in 4 h and the monomer ratio to the internal standard was measured by ^{1}H NMR. The conversion was determined as 79.0% by using the ratio before polymerization.

For PPM, 5.0 mL of the polymerization solution was transferred to a new glass tube containing phenothiazine (inhibitor, 20.0 mg). The solvent [i.e., $(CH_2Cl)_2$] was evaporated under reduced pressure, followed by the addition of 5.0 ml anhydrous THF. Isopropyl amine (0.43 mL, 5.0 mmol) was added to the mixture and the resultant mixture was immersed in an oil bath at 60 ˚C and kept stirring for 24 h. The resulting solution was poured to 10 fold excess volume of methanol and the polymer was precipitated. The precipitation was centrifuged and washed with methanol after decantation. Finally, the product was dried under vacuum. This process yielded 25.7 mg of polyNIPAM (58 % yield). The ^{1}H NMR spectrum of thus-obtained product was conducted in DMSO-d_6 at 150 ˚C, and the dyad tacticity was determined by ^{1}H NMR: $m : r$ = 94 : 6. The molecular weight and the molecular weight distribution could not be measured by SEC due to its poor solubility. For the polymerization at 30 ˚C, V-70 was used as an initiator instead of AIBN. Due to the differing tacticity and solubility of the obtained polyNIPAM, purification differed based on the solubility of the polymer in methanol.

Product via polymerization of **Me**-BTDAm at 60 ˚C and subsequent PPM with isopropyl amine

Monomer conversion: 56%

Purification: Dialysis with methanol

Yield: 12.6 mg, 40%

Tacticity: $m : r$ = 70 : 30.

Product via polymerization of **Me**-BTDAm at 30 ˚C and subsequent PPM with isopropyl amine

Monomer conversion: 71%

Purification: Dialysis with methanol

Yield: 28.6 mg, 71%

Tacticity: *m* : *r* = 77 : 23.

Product via polymerization of ***i*Pr**-BTDAm at 60 ˚C and subsequent PPM with isopropyl amine

Monomer conversion: 62%

Purification: Dialysis with methanol

Yield: 32.4 mg, 92%

Tacticity: *m* : *r* = 80 : 20.

Product via polymerization of ***i*Pr**-BTDAm at 30 ˚C and subsequent PPM with isopropyl amine

Monomer conversion: 70%

Purification: Dialysis with methanol

Yield: 32.8 mg, 83%

Tacticity: *m* : *r* = 83 : 17.

Product via polymerization of **Ph**-BTDAm at 60 ˚C and subsequent PPM with isopropyl amine

Monomer conversion: 79%

Purification: Precipitating into methanol and washed with methanol

Yield: 25.7 mg, 58%

Tacticity: *m* : *r* = 94 : 6.

Product via polymerization of **Ph**-BTDAm at 30 ˚C and subsequent PPM with isopropyl amine

Monomer conversion: 87%

Purification: Precipitating into methanol and washed with methanol

Yield: 45.5 mg, 92%

Tacticity: *m* : *r* = 95 : 5.

Polymerization at 0 ˚C or -40 ˚C

General Procedure for Polymerization and Transformation into Poly(*N*-isopropylacrylamide). The solubility of the obtained polyNIPAM in methanol varied depending on tacticity, and thus the purification method was modified according to the solubility. A general procedure follows:

In a Schlenk tube, **Ph**-BTDAm (0.18 g, 0.55 mmol), AIBN (1.8 mg, 11.0 μmol), $(CH_2Cl)_2$ (5.3 ml), and a small portion of tetralin (internal standard) were added in this order at room temperature under dry argon to give the polymerization solution with the following concentrations: $[$**Ph**-BTDAm$]_0/[AIBN]_0$ = 100/2 mM. The tube was immersed in chilled methanol at 0 ˚C and a UV-LED lamp (LDL-71X12UV3-365-N, λ = 365 nm, 7.6 W, CCS Inc., Kyoto, Japan) was set at a distance of approximately 10 cm from the tube. The polymerization was initiated by the UV irradiation. After 24 h irradiation at 0˚C, the irradiation was stopped, and a small portion of the polymerization solution was diluted with $CDCl_3$ for determination of conversion by ^{1}H NMR similar to the polymerization at 60˚C: conversion >99%. To a glass tube, 5.0 mL of the polymerization solution was added, which was further evaporated under reduced pressure, followed by the addition of 5.0 ml anhydrous THF. Isopropyl amine (0.43 mL, 5.0 mmol) was slowly added to the mixture and the resultant mixture was immersed in an oil bath at 60 ˚C and kept stirring for 24 h. The resulting solution was purified by precipitating into 10 fold excess volume of methanol. It was centrifuged and after decantation the sediment was washed with methanol, which was further dried under vacuum. This process yielded 40.3 mg of poly(*N*-isopropylacrylamide). The ^{1}H NMR spectrum of thus-obtained product was conducted in DMSO-d_6 at 150 ˚C, and the dyad tacticity was determined by ^{1}H NMR: $m : r$ = 97 : 3. The molecular weight and the molecular weight distribution could not be measured by SEC due to its poor solubility. The polymerization at -40 ˚C was carried out in the same way. For transamidation of poly(**Me**-BTDAm) and poly(***i*Pr**-BTDAm), 20.0 mg of phenothiazine (radical inhibitor) was added after 24h of UV irradiation.

Product via polymerization of **Me**-BTDAm at 0 ˚C and subsequent PPM with isopropyl amine

Monomer conversion: 90%

Purification: Dialysis with methanol

Yield: 44.0 mg, 86%

Tacticity: $m : r$ = 82 : 18.

Product via polymerization of **Me**-BTDAm at -40 ˚C and subsequent PPM with isopropyl amine

Monomer conversion: 99%

Purification: Dialysis with methanol

Yield: 45.0 mg, 80%

Tacticity: *m* : *r* = 83 : 17.

Product via polymerization of ***i*Pr**-BTDAm at 0 ˚C and subsequent PPM with isopropyl amine

Monomer conversion: 88%

Purification: Dialysis with methanol

Yield: 39.3 mg, 79%

Tacticity: *m* : *r* = 89 : 11.

Product via polymerization of ***i*Pr**-BTDAm at -40 ˚C and subsequent PPM with isopropyl amine

Monomer conversion: 99%

Purification: Dialysis with methanol

Yield: 49.5 mg, 88%

Tacticity: *m* : *r* = 95 : 5.

Product via polymerization of **Ph**-BTDAm at 0 ˚C and subsequent PPM with isopropyl amine

Monomer conversion: 99%

Purification: Precipitating into methanol and washed with methanol

Yield: 40.3 mg, 71%

Tacticity: *m* : *r* = 97 : 3.

Product via polymerization of **Ph**-BTDAm at -40 ˚C and subsequent PPM with isopropyl amine

Monomer conversion: 99%

Purification: Precipitating into methanol and washed with methanol

Yield: 46.5 mg, 82%

Tacticity: *m* : *r* = 99 : 1.

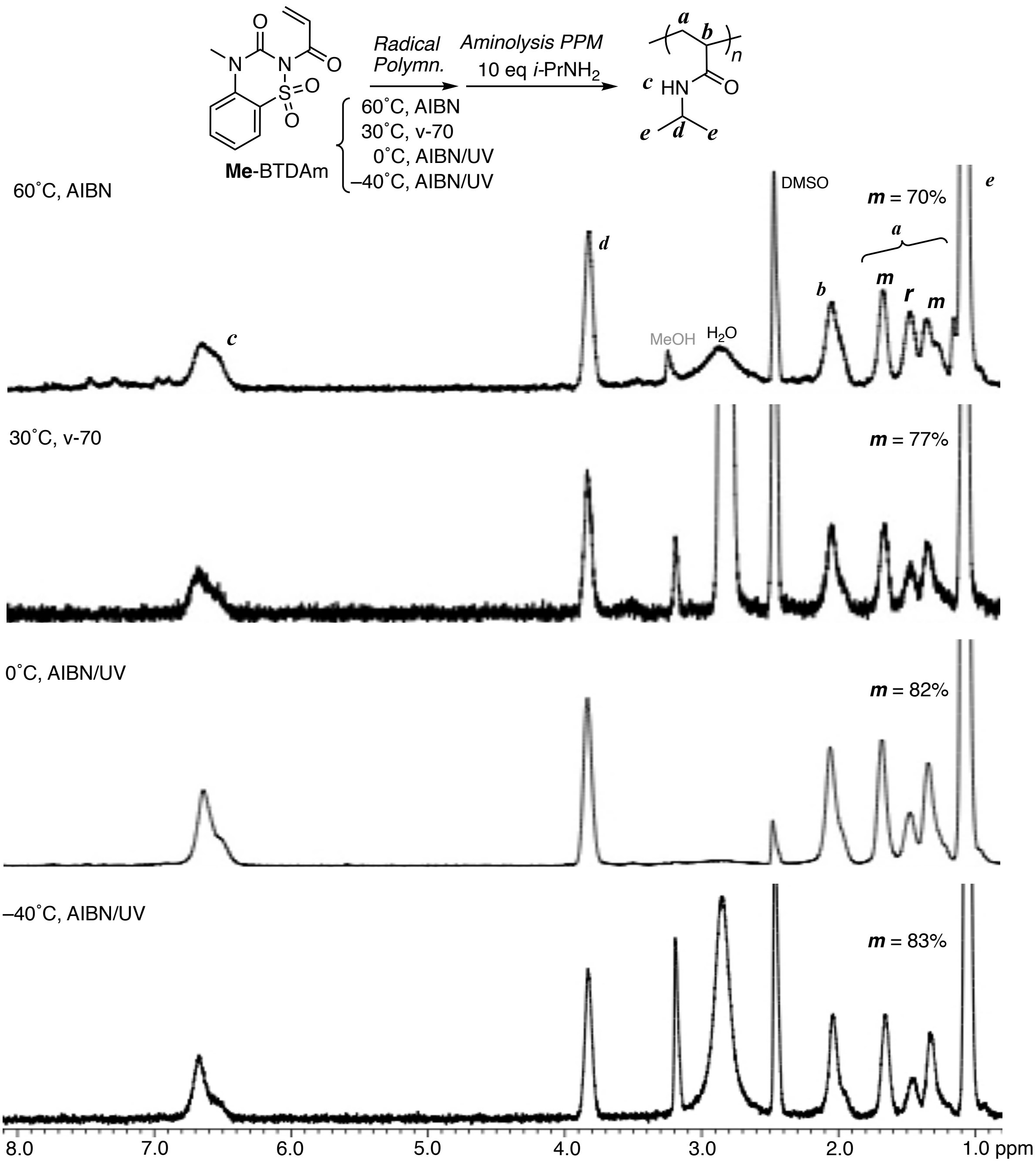

Figure S1. Radical polymerizations of **Me**-BTDAm at different temperatures and aminolysis PPM with *i*PrNH2 for evaluation of isotacticity (*m*) of resultant polyNIPAM (0.8-8.1 ppm).

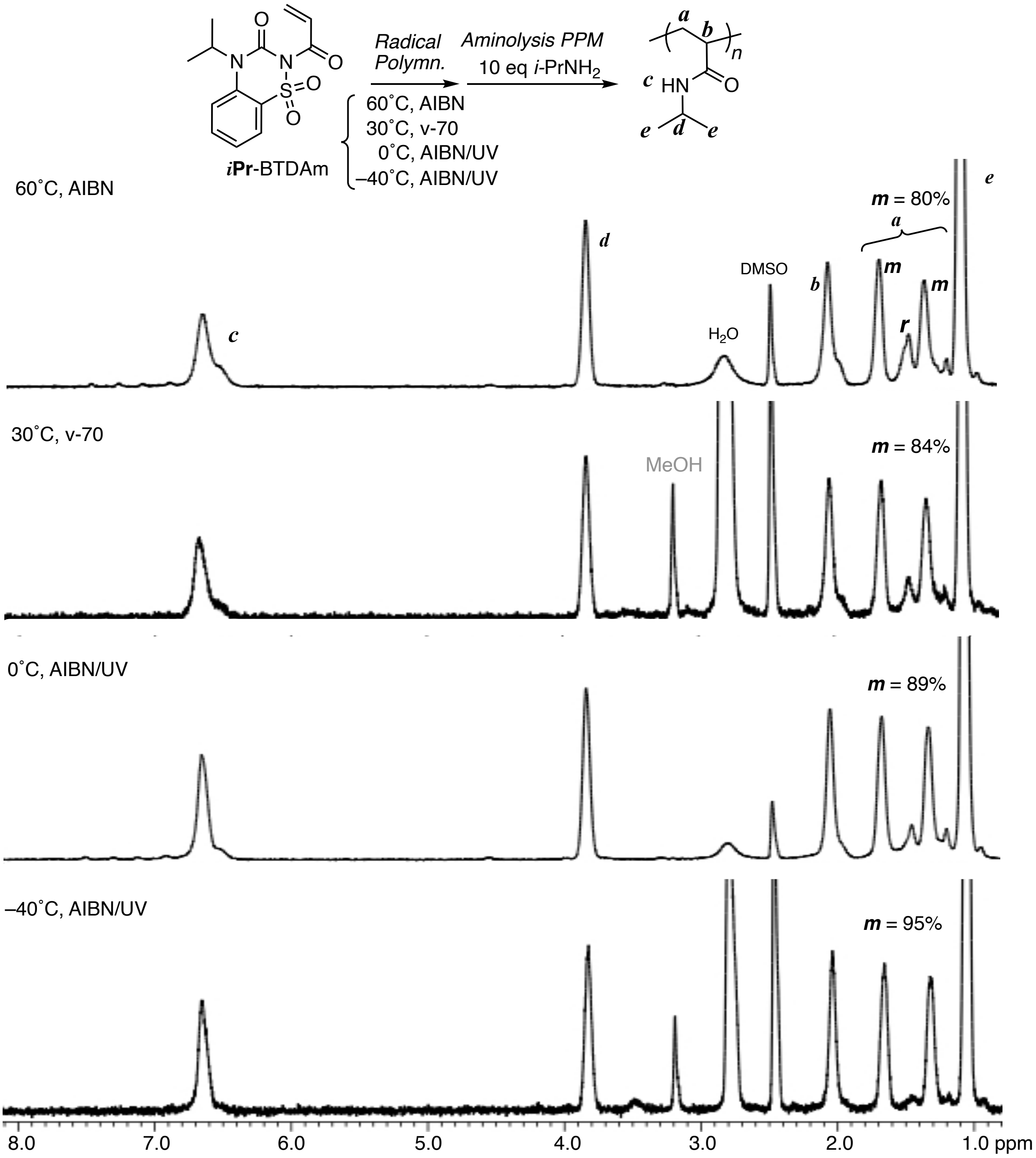

Figure S2. Radical polymerizations of ***i*Pr**-BTDAm at different temperatures and aminolysis PPM with *i*PrNH$_2$ for evaluation of isotacticity (*m*) of resultant polyNIPAM (0.8-8.1 ppm).

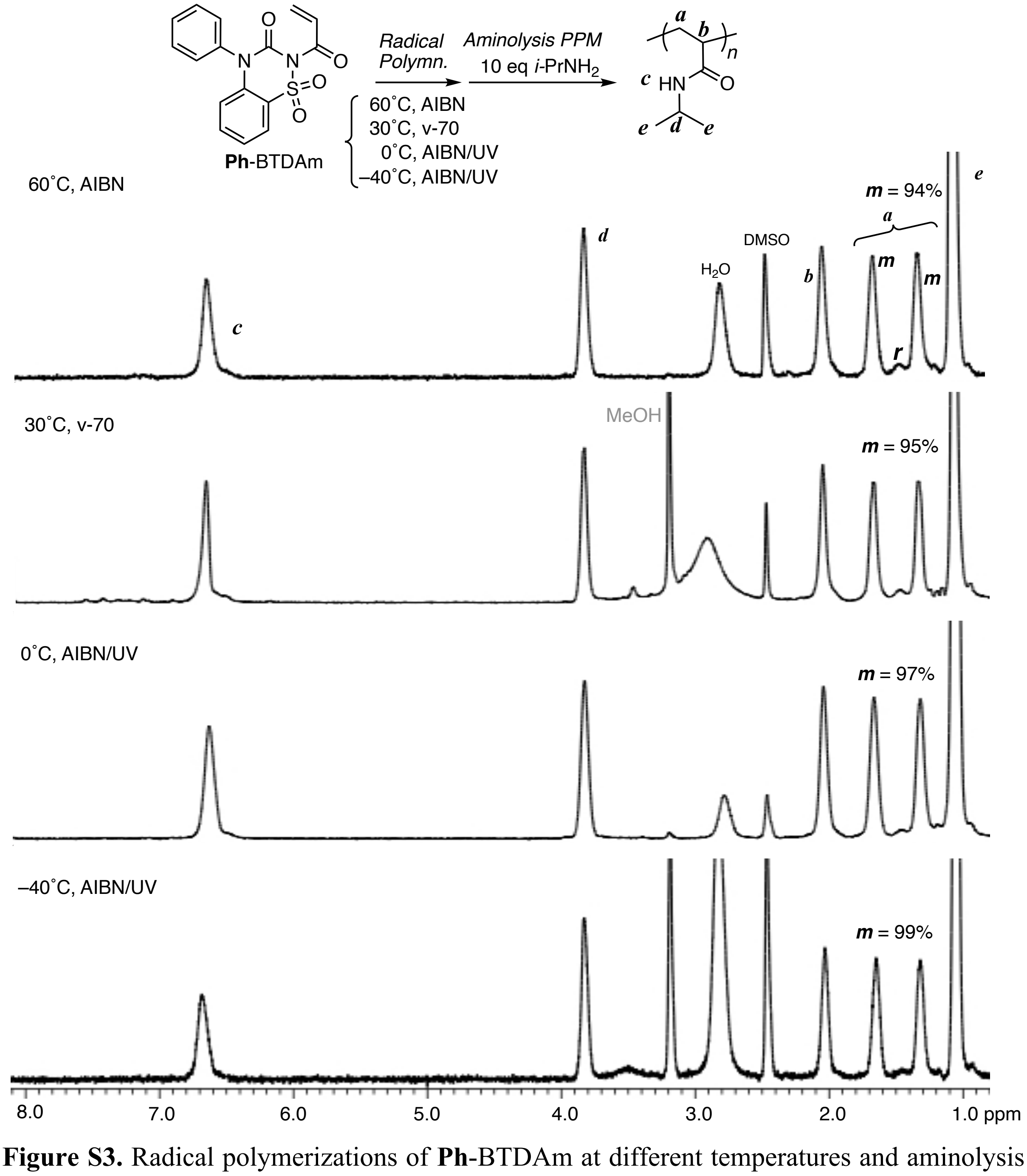

Figure S3. Radical polymerizations of **Ph**-BTDAm at different temperatures and aminolysis PPM with *i*PrNH$_2$ for evaluation of isotacticity (*m*) of resultant polyNIPAM (0.8-8.1 ppm).

3.2. Syntheses of Isotactic Polyacrylamides with PhBTDAm

Radical Polymerization of **Ph**-BTDAm at -40 ˚C in Larger Scale

In a round bottom flask, **Ph**-BTDAm (3.28 g, 10.0 mmol), AIBN (32.8 mg, 0.2 mmol) and $(CH_2Cl)_2$ (97.0 ml) were added in this order under dry argon to give the polymerization solution with the following concentrations: $[$**Ph**-BTDAm$]_0$/$[AIBN]_0$ = 100/2 mM. The flask was immersed in methanol chilled at -40 ˚C and a UV-LED lamp (LDL-71X12UV3-365-N, λ = 365 nm, 7.6 W, CCS Inc., Kyoto, Japan) was set at a distance of approximately 10 cm from the flask. The polymerization was initiated upon UV irradiation. After 24 h, the irradiation was stopped for termination of the polymerization and the resulting polymer solution was stocked at -20˚C. The part of the stocked solution underwent the subsequent aminolysis transformation (see below).

Aminolysis Transformation with Various Amine Compounds for Synthesis of Isotactic Poly(acrylamide)s

According to the nucleophilicity of amine compounds and the solubility of the obtained polymers, the condition for aminolysis transformation and the polymer purification were modified. The seven methods (Method A-G) were used as follows:

Method A (for 1 and 5-12): To a glass tube, 5.0 mL of the stocked solution after radical polymerization of **Ph**-BTDAm at -40˚C was added. The solution was evaporated under reduced pressure, followed by the addition of 5.0 ml anhydrous THF and amine compound (5.0 mmol). The solution was immersed in an oil bath at 60 ˚C and kept stirring for 24 h. The resulting solution was purified by precipitating into MeOH. The precipitate was washed with MeOH and dried under vacuum.

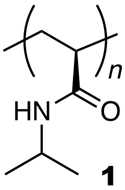

1

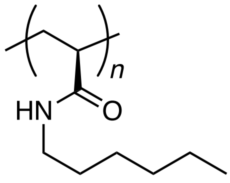

5

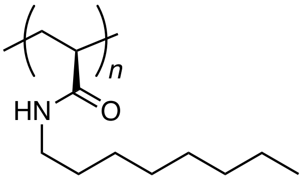

6

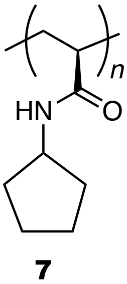

7

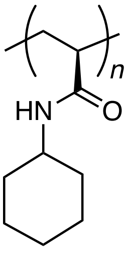

8

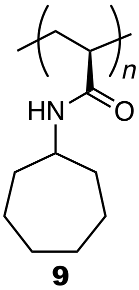

9

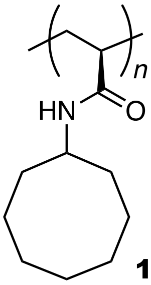

10

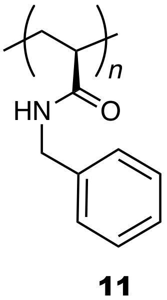

11

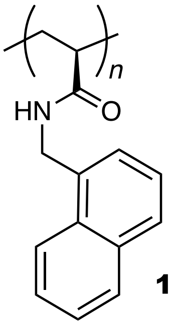

12

Method B (for 2-4, 13-14 and 16-17): The aminolysis reaction was the same as Method A. The resultant polymers were soluble in MeOH and thus the resulting solution was purified by dialysis with MeOH (3 times replacement of the solvent and stirring for longer than 4 hours for each). The solution was dried under vacuum.

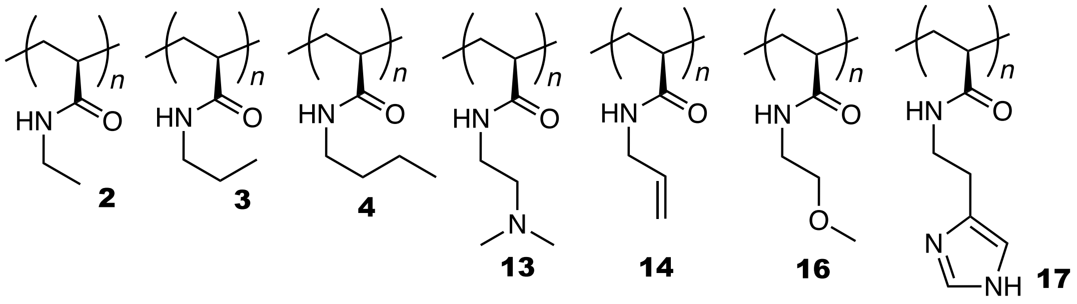

Method C (for 15): To a glass tube, 5.0 mL of the stocked solution after radical polymerization of **Ph**-BTDAm at -40˚C was added. The solution was evaporated under reduced pressure, followed by the addition of 5.0 ml anhydrous DMSO and 2-aminoethanol (5.0 mmol). The solution was immersed in an oil bath at 60 ˚C and kept stirring for 24 h. The resultant polymer (**15**) was soluble in MeOH and thus was purified in the same way as Method B (dialysis with MeOH).

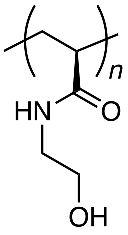
15

Method D (for 18): To a glass tube, 5.0 mL of the stocked solution after radical polymerization of **Ph**-BTDAm at -40˚C was added. The solution was evaporated under reduced pressure, followed by the addition of 5.0 ml anhydrous DMSO. Glycinamide hydrochloride (1.0 mmol) and triethylamine (2.5 mmol) was added to the solution. The resultant solution was immersed in an oil bath at 60 ˚C and kept stirring for 24 h. The polymer (**18**) was purified in the same way as Method A (precipitation in MeOH).

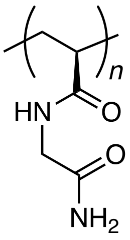
18

Method E (for 19): To a glass tube, 5.0 mL of the stocked solution after radical polymerization of **Ph**-BTDAm at -40˚C was added. The solution was evaporated under reduced pressure, followed by the addition of 5.0 ml 1,2-dimethoxyethane and morpholine (5.0 mmol). The solution was immersed in an oil bath at 80 ˚C and kept stirring for 72 h. The polymer (**19**) was purified in the same way as Method A (precipitation in MeOH).

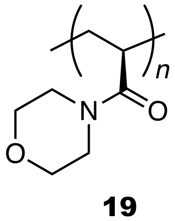
19

Method F (for 20): To a glass tube, 5.0 mL of the stocked solution after radical polymerization of **Ph**-BTDAm at -40˚C was added. The solution was evaporated under reduced pressure, followed by the addition of 5.0 ml anhydrous THF and diethylamine (5.0 mmol). The solution was immersed in an oil bath at 60 ˚C and kept stirring for 42 h. The resultant polymer (**20**) was soluble in MeOH and thus was purified in the same way as Method B (dialysis with MeOH).

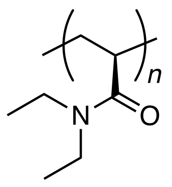

20

Method G (for 21): To a glass tube, 5.0 mL of the stocked solution after radical polymerization of **Ph**-BTDAm at -40˚C was added. The solution was evaporated under reduced pressure, followed by the addition of 5.0 ml 1,2-dimethoxyethane and dibutylamine (5.0 mmol). The solution was immersed in an oil bath at 80 ˚C and kept stirring for 42 h. The polymer (**21**) was purified in the same way as Method A (precipitation in MeOH).

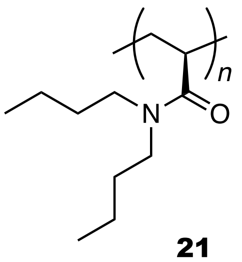

21

3.3. Syntheses of Atactic Polyacrylamides

General Procedure

Most of atactic poly(*N*-alkylacrylamide)s were synthesized as follows. In a Schlenk tube, *N*-alkylacrylamide (3.0 mmol), AIBN (4.9 mg, 30.0 µmol), dioxane (2.5 ml), and a small portion of tetralin (internal standard) were added in this order at room temperature under dry argon to give the polymerization solution with the following concentrations: $[N\text{-alkylacrylamide}]_0/[\text{AIBN}]_0$ = 1000/10 mM. The tube was then immersed in an oil bath at 60 ˚C. A small portion of the polymerization solution was diluted with $CDCl_3$ after 24 h, and the monomer conversions were determined by ^{1}H NMR, where the integration of the peak area from the olefinic protons relative to those of tetralin as an internal standard was compared with that of before heating The resulting solution was purified by precipitation into diethyl ether (twice), and the precipitate was collected by centrifugation and then dried under vacuum to yield atactic polymers. Poly(*N*-hydroethyl acrylamide) was purified by methanol dialysis instead of the precipitation into diethyl ether. For the monomers, **NaphAm** and **cHexAm**, the polymerizations were performed under diluted condition due to lower solubilities: $[\text{monomer}]_0/[\text{AIBN}]_0$ = 500/10 mM. **EtAm** was polymerized in ethanol instead of dioxane. In most cases, the molecular weight and the molecular weight distribution were measured by SEC in LiBr-contained DMF (10 mM) as an eluent. In the case of poly(OctAm), poly(cPenAm), poly(cHexAm), poly(cHepAm), poly(cOctAm) and poly(BzAm), they were measured by SEC in THF. As for poly(HEAm), it was difficult to measure the molecular weight by SEC due to absorption to column. ^{1}H NMR spectrum is shown in Figure S21-33, S35, S39-40.

N-Acryloyl glycinamide (**AcGAm**) was polymerized according to the literature[3]: In a Schlenk tube, **AcGAm** (2.0 mmol), AIBN (3.2 mg, 20.0 µmol), and DMSO (10.0 ml) were added in this order at room temperature under dry argon to give the polymerization solution with the following concentrations: $[\textbf{AcGAm}]_0/[\text{AIBN}]_0$ = 200/2 mM. The tube was then immersed in an oil bath at 70 ˚C for 2 h. The resulting solution was purified by precipitating into methanol twice. The precipitate was collected by centrifugation and dried under vacuum at 80 ˚C. ^{1}H NMR spectrum is shown in Figure S38.

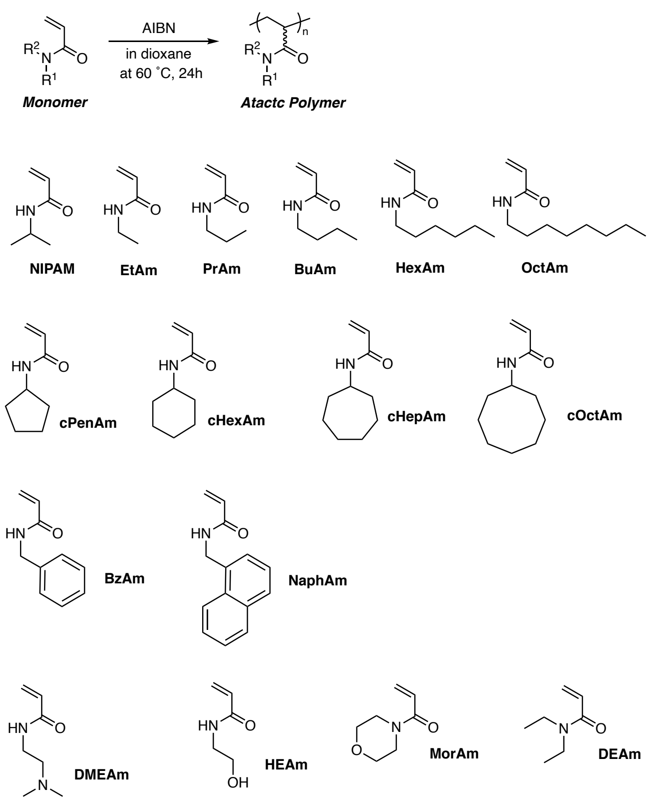

Monomer	$[\text{Monomer}]_0$ (M)	Monomer Conv.(%)	M_n (Kg/mol)	*Đ*
**NIPAM**	1.0	96.3	37.9	2.70
**EtAm**	1.0	95.1	12.4	3.08
**PrAm**	1.0	97.1	43.6	2.52
**BuAm**	1.0	95.8	28.0	2.65
**HexAm**	1.0	98.6	40.6	1.69
**OctAm**	1.0	97.1	28.4	2.40
**cPenAm**	1.0	96.9	10.8	1.70
**cHexAm**	0.5	95.5	25.9	1.79
**cHepAm**	1.0	96.8	25.9	1.79
**cOctAm**	1.0	98.6	31.1	2.56
**BzAm**	1.0	89.6	54.5	1.81
**NaphAm**	0.5	34.8	15.2	1.97
**DMEAm**	1.0	96.6	10.0	1.81
**HEAm**	1.0	>99	n.d.	n.d.
**MorAm**	1.0	>99	34.1	2.81
**DEAm**	1.0	98.7	22.0	3.02

Figure S4. Characterizations of atactic poly(*N*-alkylacrylamide)s.

4. DSC Thermograms of Isotactic and Atactic Polyacrylamides

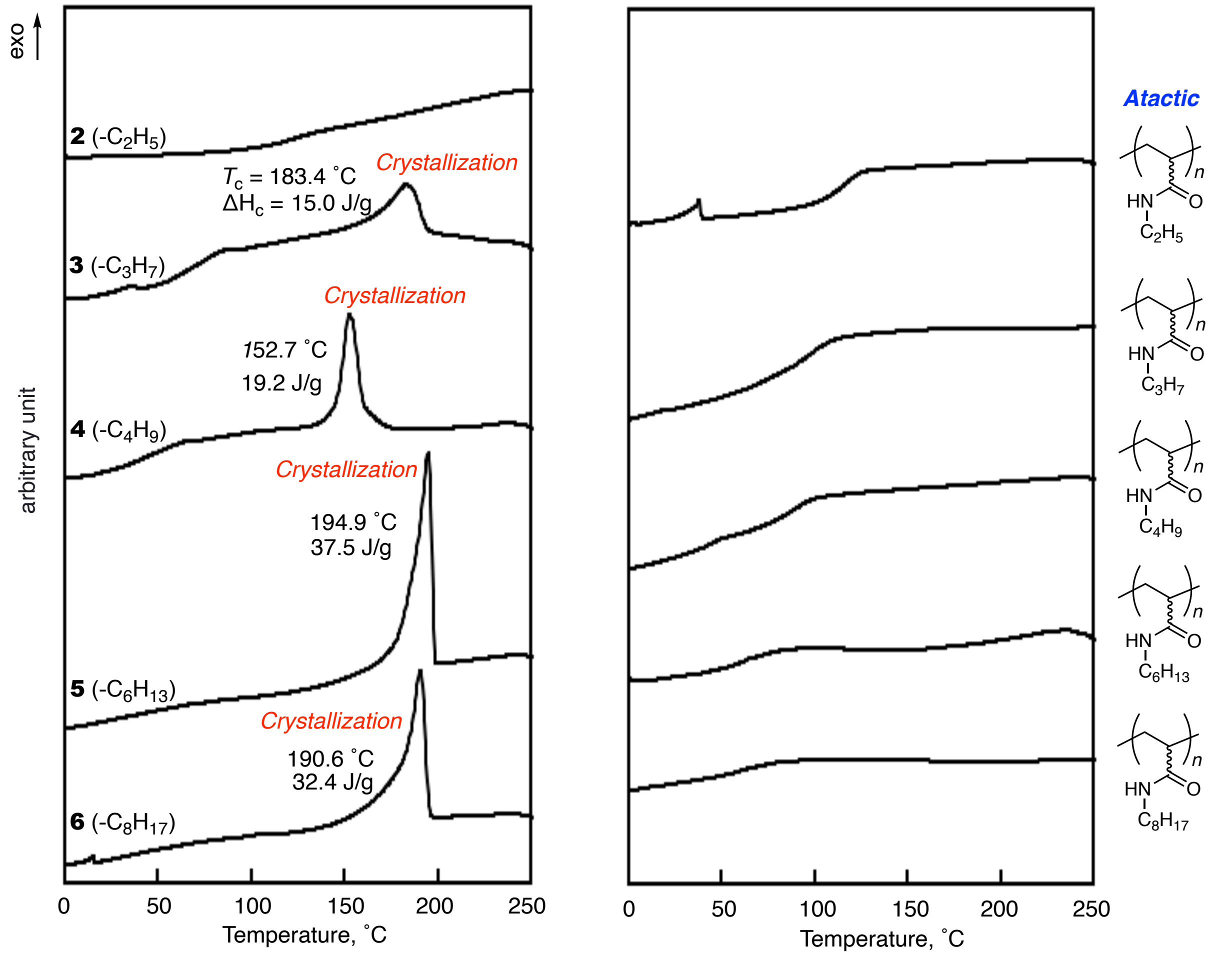

Figure S5. DSC thermograms (1st cooling) of isotactic polyacrylamides (**2**-**6**) carrying linear side chains in comparison with those of atactic polymers. The 2nd heating profiles are shown in Figure 4 in main text.

5. XRD of Isotactic Polyacrylamides with Linear Side Chains (vs Atactic Counterparts)

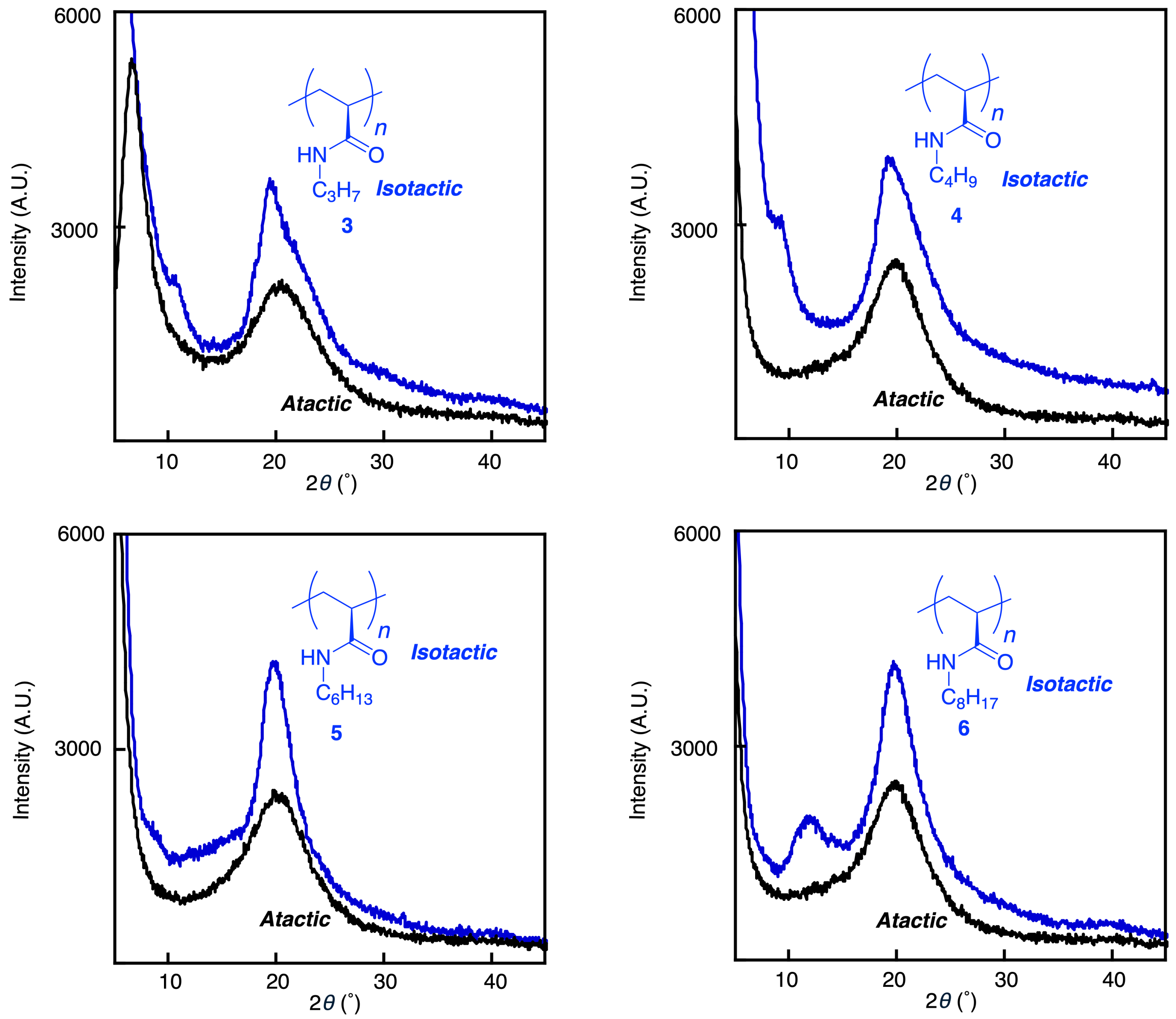

Figure S6. XRD of the isotactic polyacrylamides with linear side chains (**3**-**6**) in comparison with those of atactic polymers.

6. Recycle of Pendant Group

5.0 mL of the stocked solution after radical polymerization of **Ph**-BTDAm at -40˚C was added to a glass tube. The solution was evaporated under reduced pressure, followed by the addition of 5.0 ml anhydrous THF and isopropylamine (0.43 mL, 5.0 mmol). The solution was then immersed in an oil bath at 60 ˚C and kept stirring for 24 h. The resulting solution was poured into 10 fold excess volume of methanol (~50 mL), leading to the precipitation of the polymer. The precipitate was collected by centrifugation and washed with methanol, yielding 46.5 mg of isotactic PolyNIPAM (yield: 82%). The supernatant was concentrated under reduced pressure, and the crude solid was dissolved in 1:1 mixture of H_2O and MeOH (5.0 mL) in the presence of an excess of $NaHCO_3$ (saturated) to convert into the sodium salt. Then, the solution was fiterer and the homogeneous solution was acidified by HCl to pH 1 to precipitate the crystallized compound. The crystal was then washed with hexane and dried under vacuum to obtain a compound (108.1 mg). The recovered compound was certainly **Ph**-BTD as supported by ^{1}H and ^{13}C NMR spectra (Figure S6 B and C) and the recovery yield was 79%.

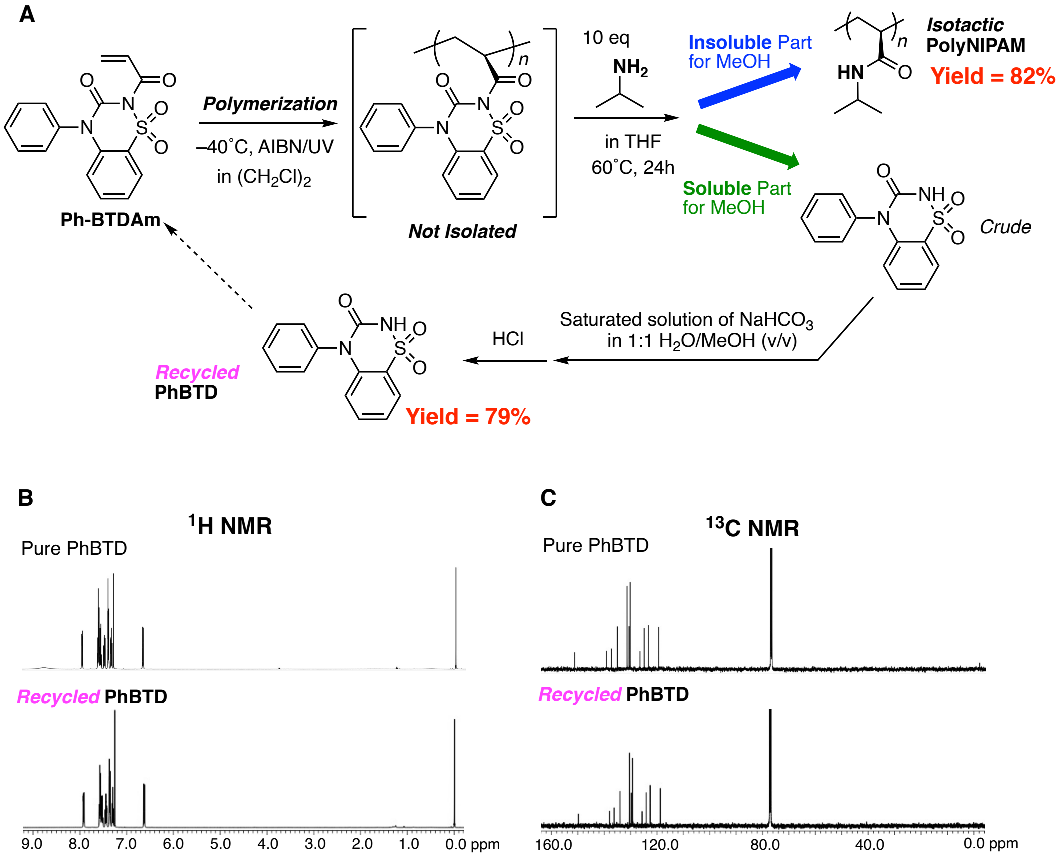

Figure S7. (A) Recovery process of the pendant group (**Ph**-BTD) from the process of isotactic polyNIPAM (i.e., radical polymerization of **Ph**-BTDAm at -40 ˚C and aminolysis transformation with isopropyl amine). (B) and (C) ^{1}H and ^{13}C NMR spectrum of recovered pendant group (**Ph**-BTD).

7. Supplementary Data

7.1. ^{1}H and ^{13}C NMR of BTDA monomers

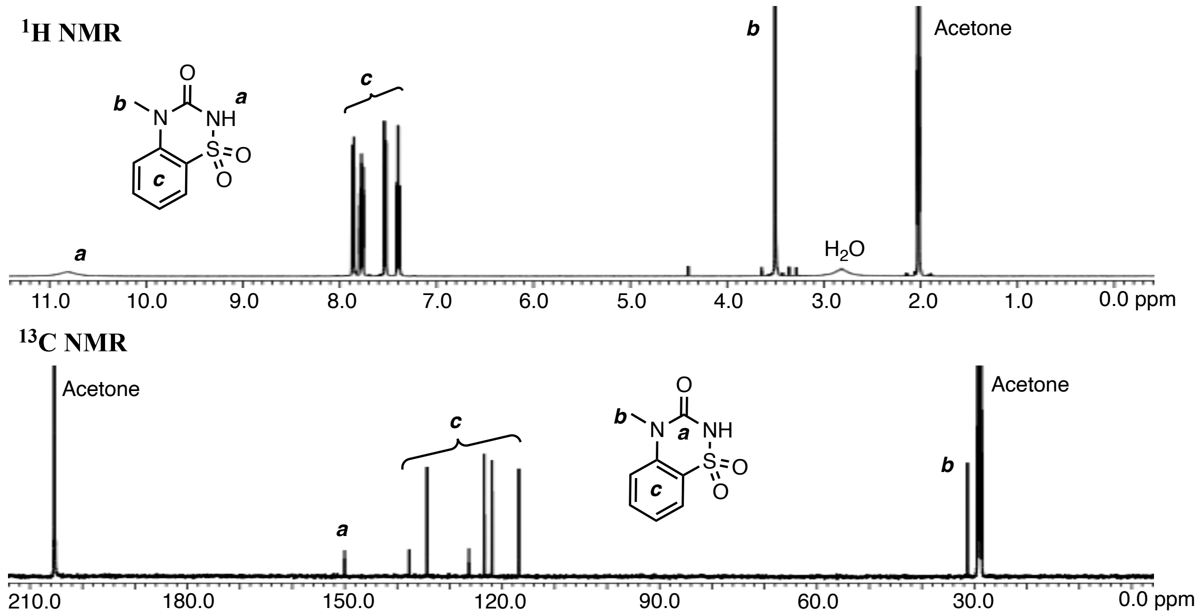

Figure S8. ^{1}H and ^{13}C NMR (room temperature) spectra of MeBTD in acetone-d_6.

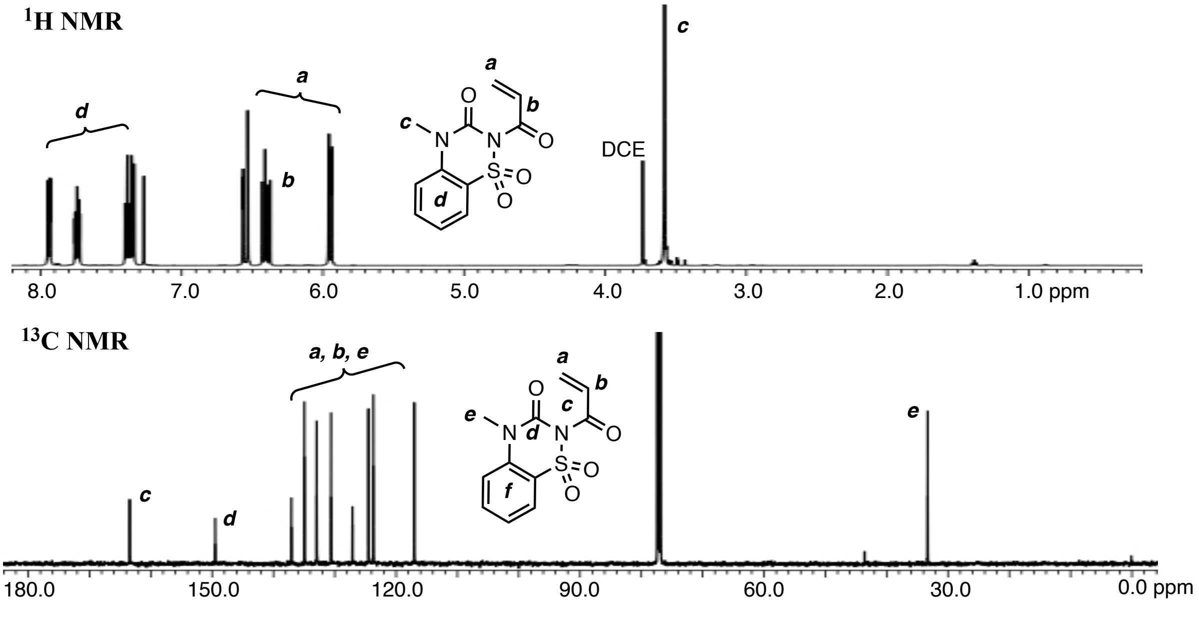

Figure S9. ^{1}H and ^{13}C NMR (room temperature) spectra of MeBTDA in $CDCl_3$,.

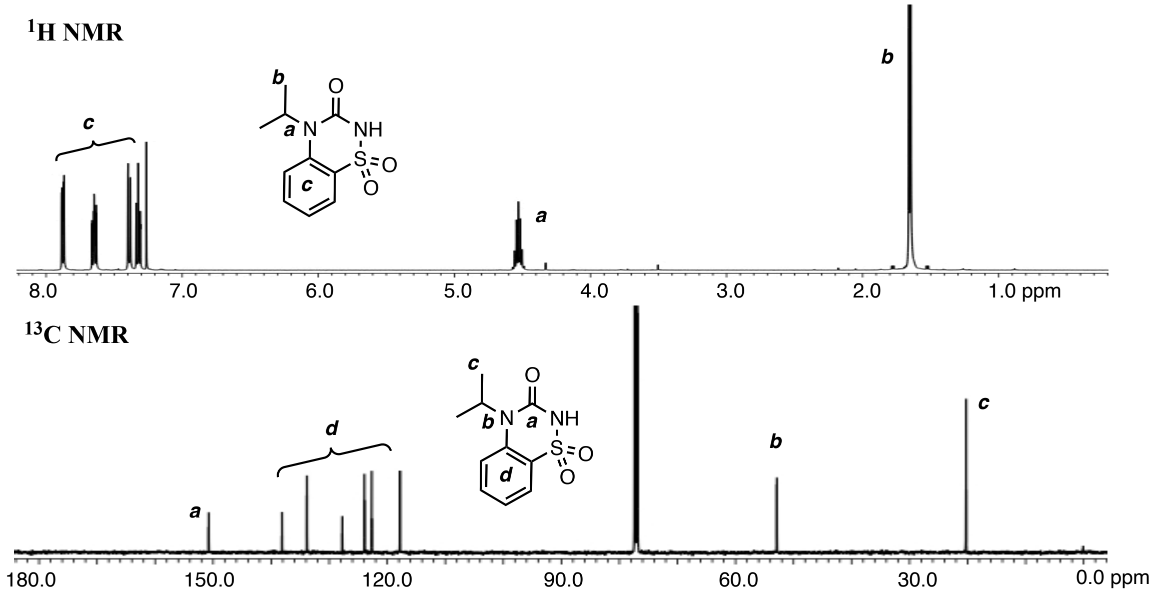

Figure S10. ^{1}H and ^{13}C NMR (room temperature) spectra of *i*PrBTD in $CDCl_3$.

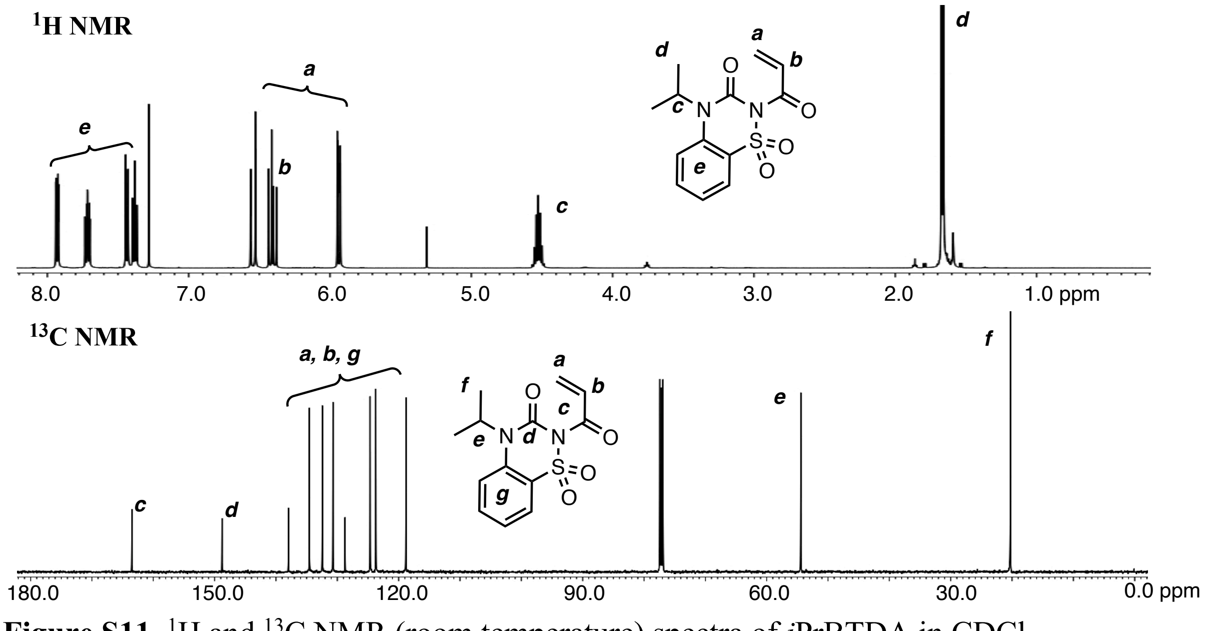

Figure S11. ^{1}H and ^{13}C NMR (room temperature) spectra of *i*PrBTDA in $CDCl_3$.

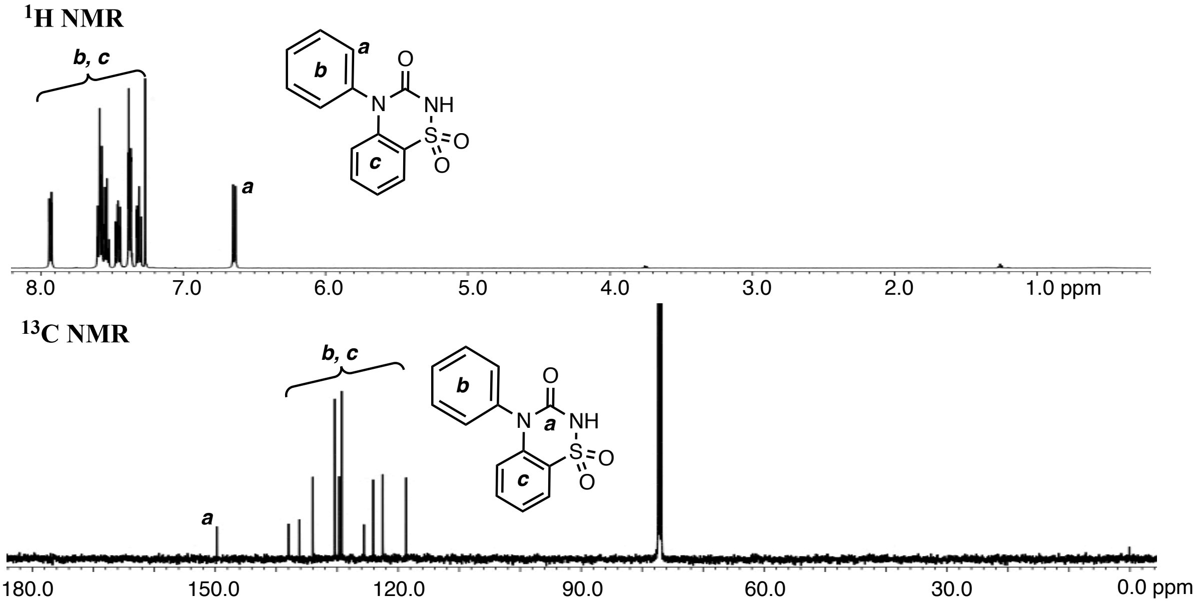

Figure S12. ^{1}H and ^{13}C NMR (room temperature) spectra of PhBTD in $CDCl_3$.

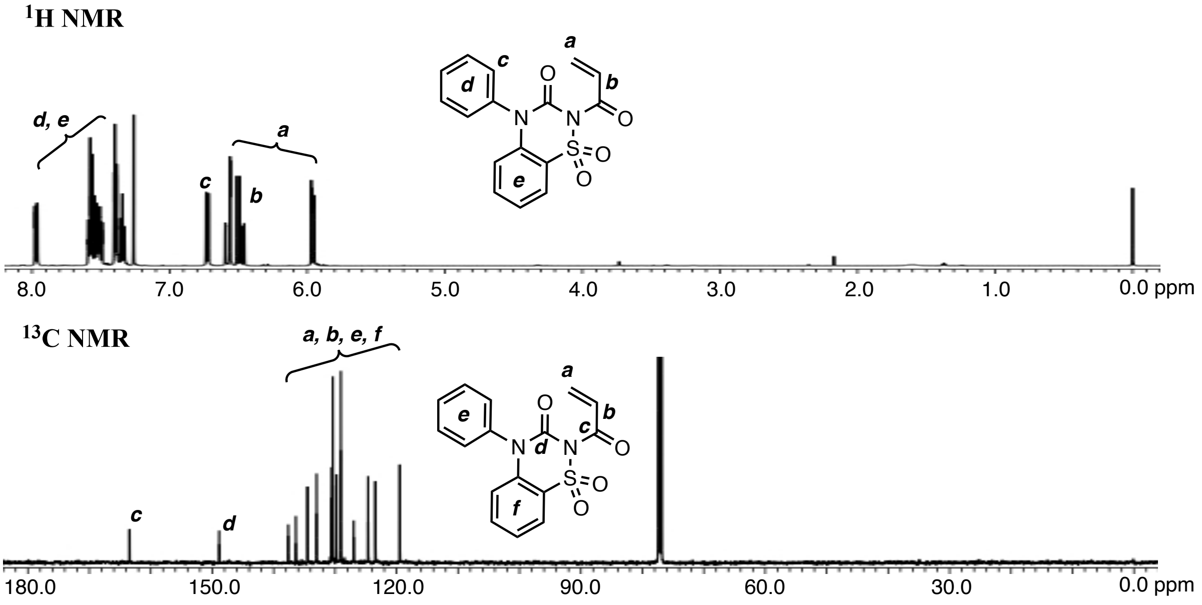

Figure S13. ^{1}H and ^{13}C NMR (room temperature) spectra of **Ph**-BTDAm in $CDCl_3$.

7.2. H and ^{13}C NMR Spectra of *N*-Alkyl Acrylamide Monomers

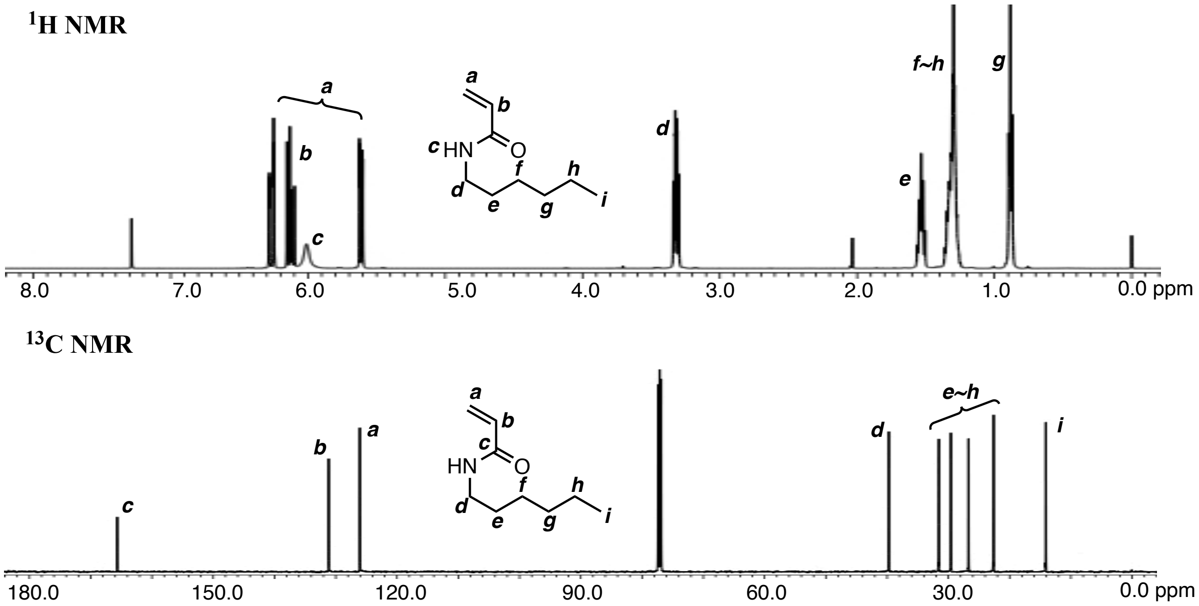

Figure S14. ^{1}H and ^{13}C NMR (room temperature) spectra of *N*-hexylacrylamide in $CDCl_3$.

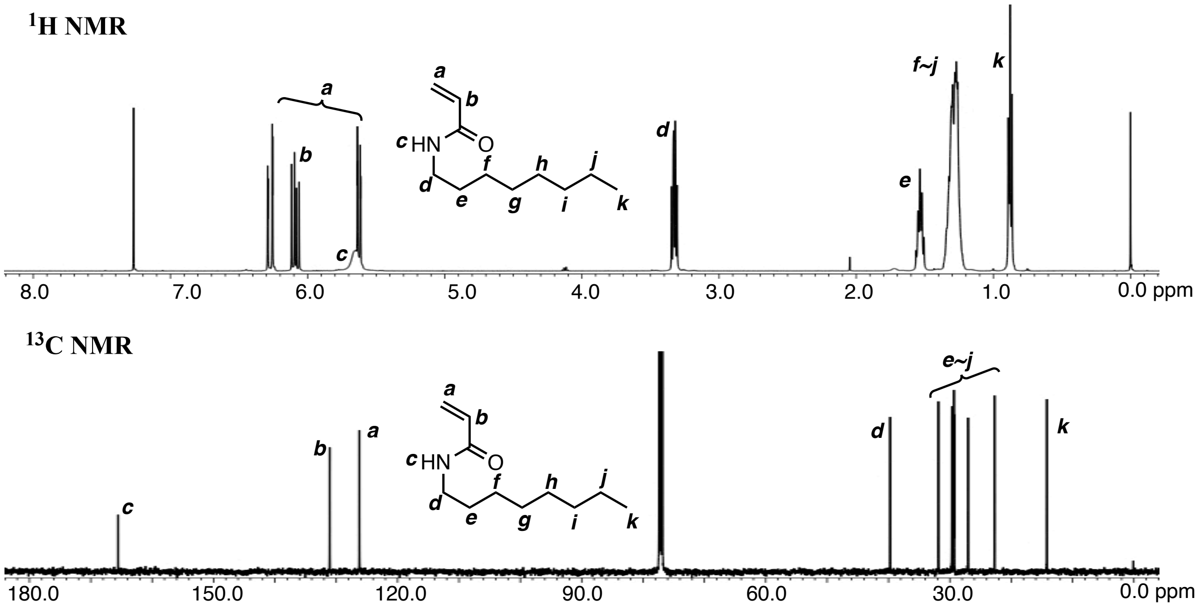

Figure S15. ^{1}H and ^{13}C NMR (room temperature) spectra of *N*-octylacrylamide $CDCl_3$.

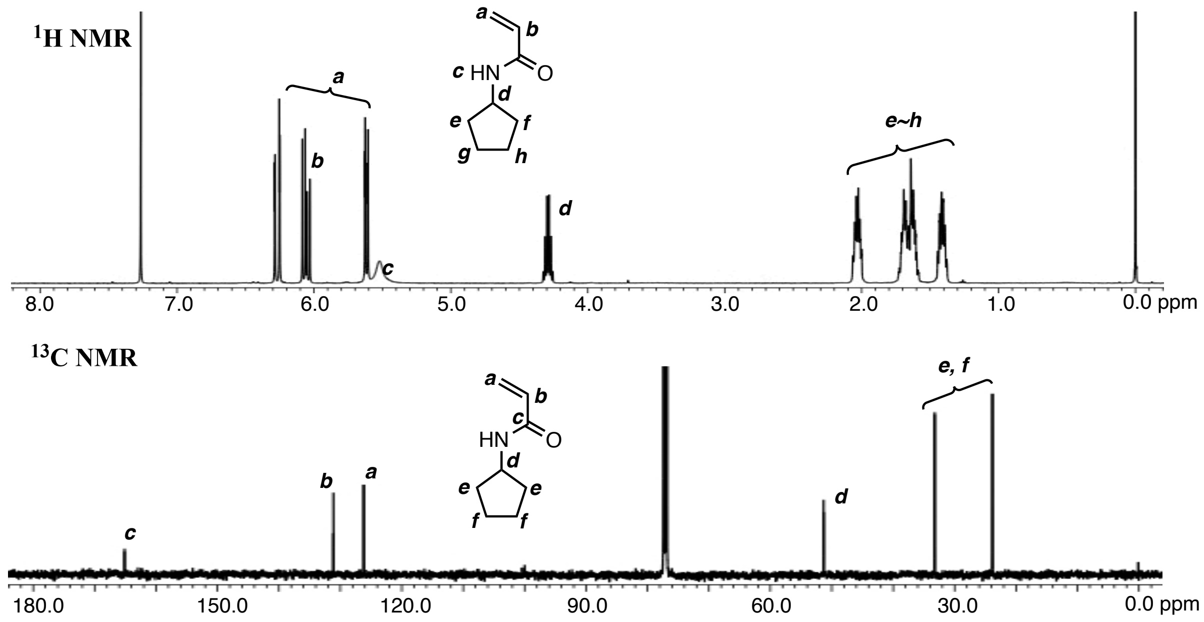

Figure S16. ^{1}H and ^{13}C NMR (room temperature) spectra of *N*-cyclopentylacrylamide in $CDCl_3$.

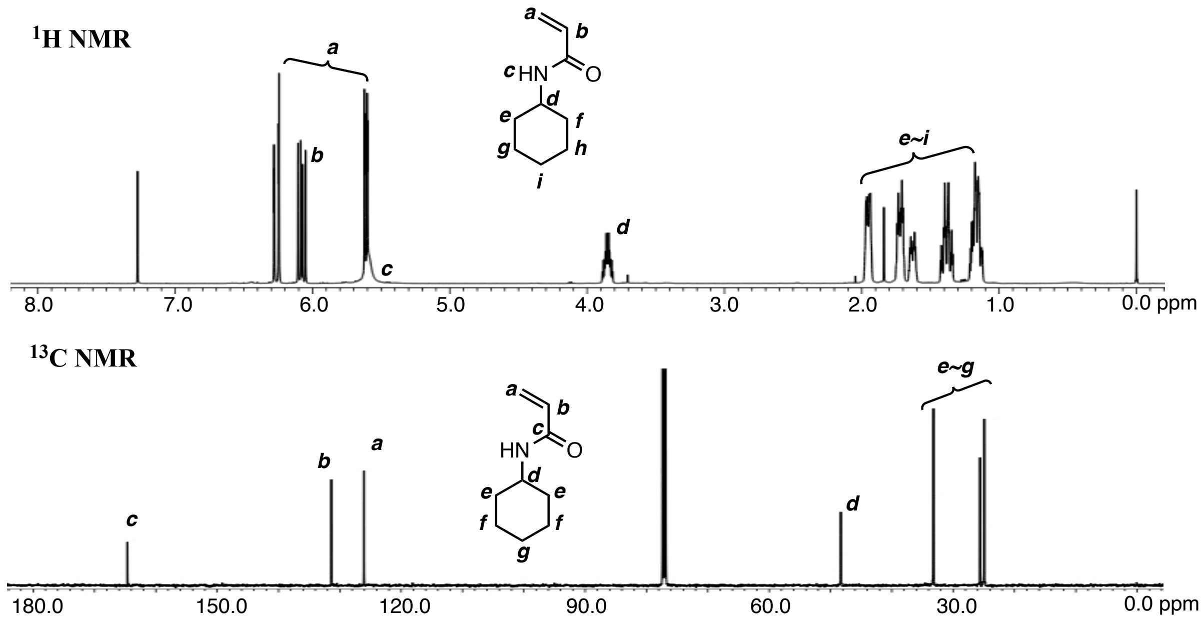

Figure S17. ^{1}H and ^{13}C NMR (room temperature) spectra of *N*-cyclohexylacrylamide in $CDCl_3$.

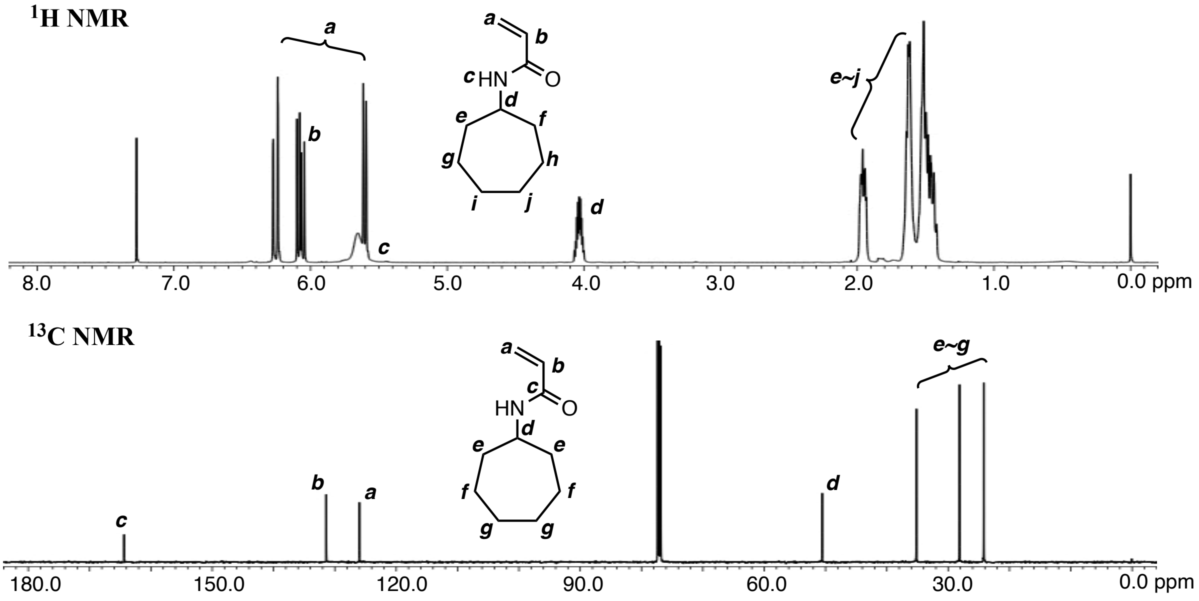

Figure S18. ^{1}H and ^{13}C NMR (room temperature) spectra of *N*-cycloheptylacrylamide in $CDCl_3$.

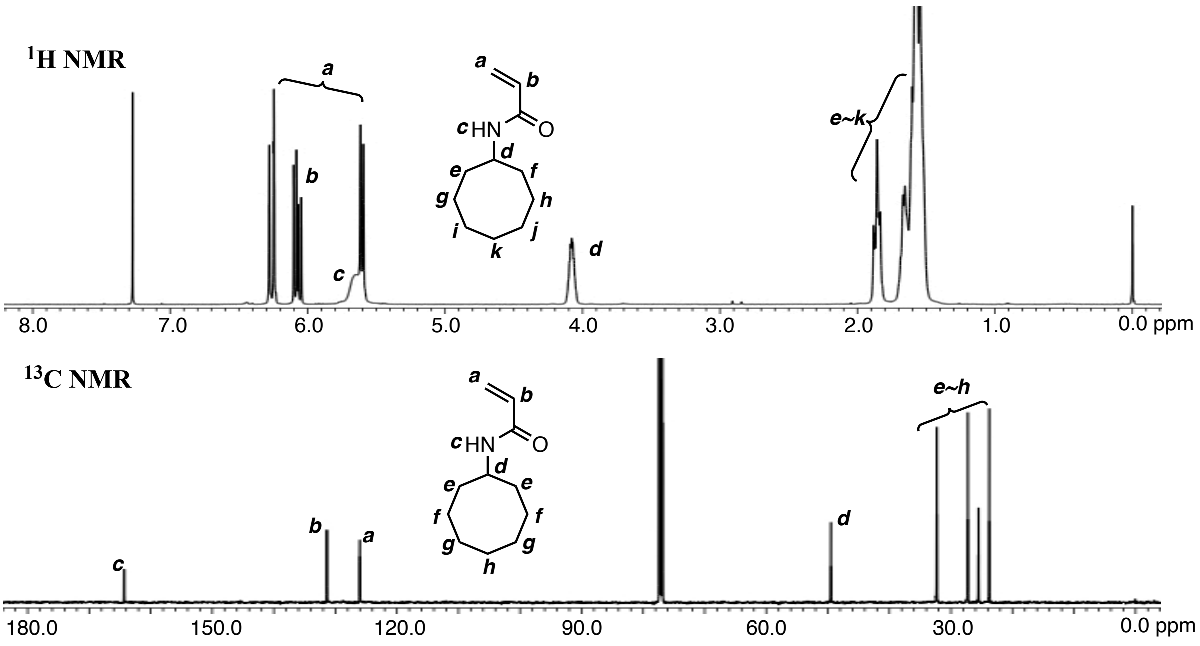

Figure S19. ^{1}H and ^{13}C NMR (room temperature) spectra of *N*-cyclooctylacrylamide in $CDCl_3$.

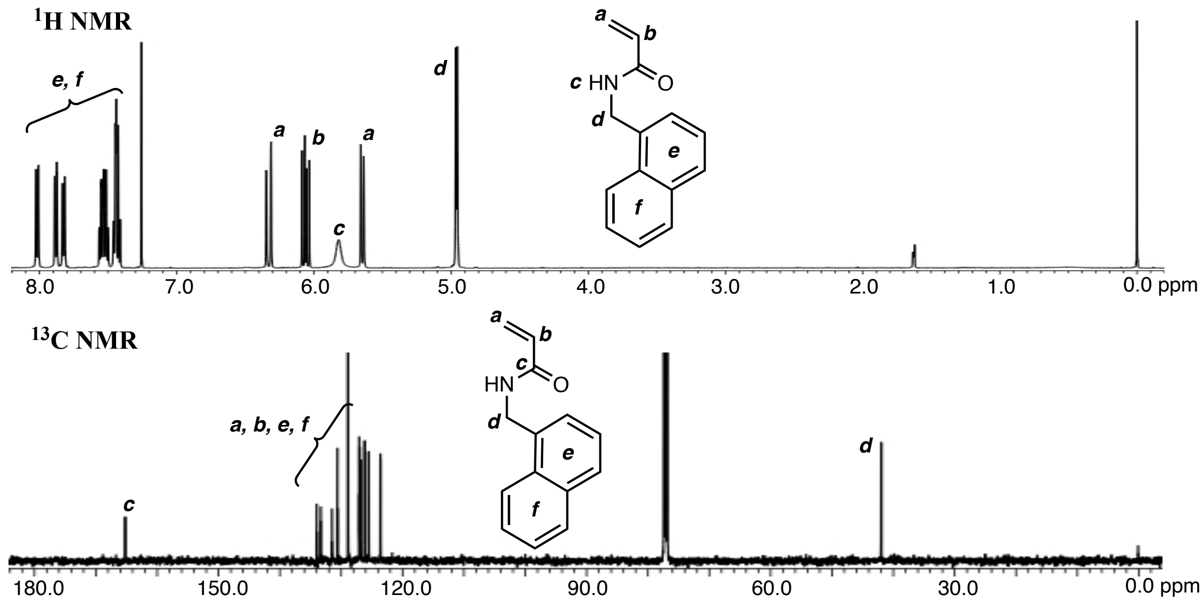

Figure S20. ¹H and ¹³C NMR (room temperature) spectra of *N*-cycloheptylacrylamide in $CDCl_3$.

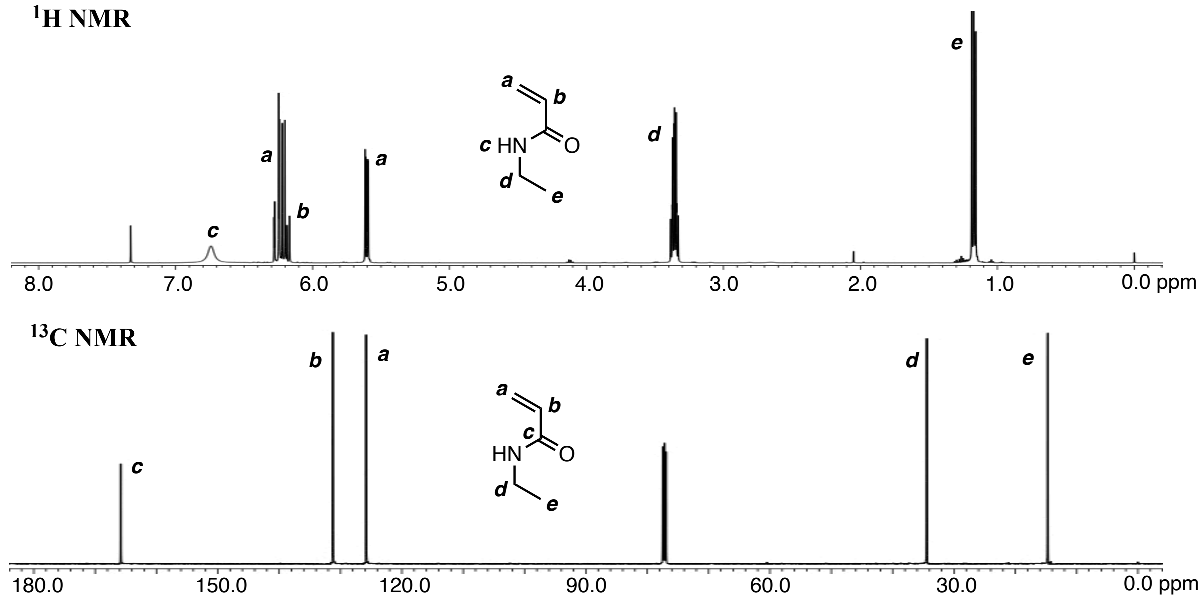

Figure S21. ¹H and ¹³C NMR (room temperature) spectra of *N*-ethylacrylamide in $CDCl_3$.

7.3. ¹H NMR Spectra of Isotactic/Atactic Polymers

In most cases, tacticity of poly(acrylamide)s was evaluated by ¹H NMR (DMSO-d_6 at 150 ˚C or $(CDCl_2)_2$ at 130 ˚C). As for poly(*N*,*N*-diethylacrylamide), CD_3OD was used as the solvent due to low solubility in DMSO-d_6 and the measurement was performed at room temperature.

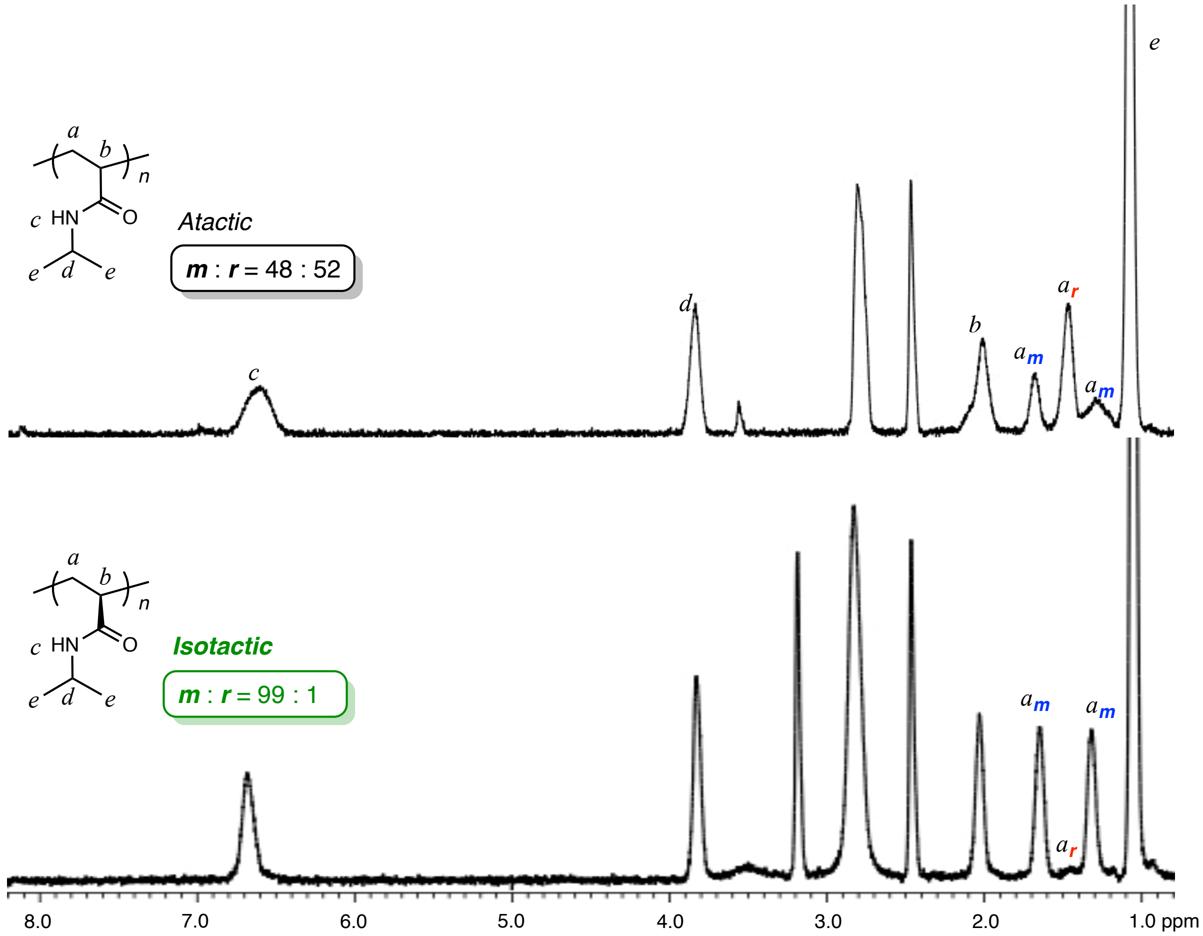

Figure S22. ¹H NMR spectrum (DMSO-d_6, 150 ˚C, lower) of the product via radical polymerization of **Ph**-BTDAm at –40˚C and subsequent aminolysis transformation with isopropylamine in comparison with that of atactic poly(*N*-isopropylacrylamide) (upper).

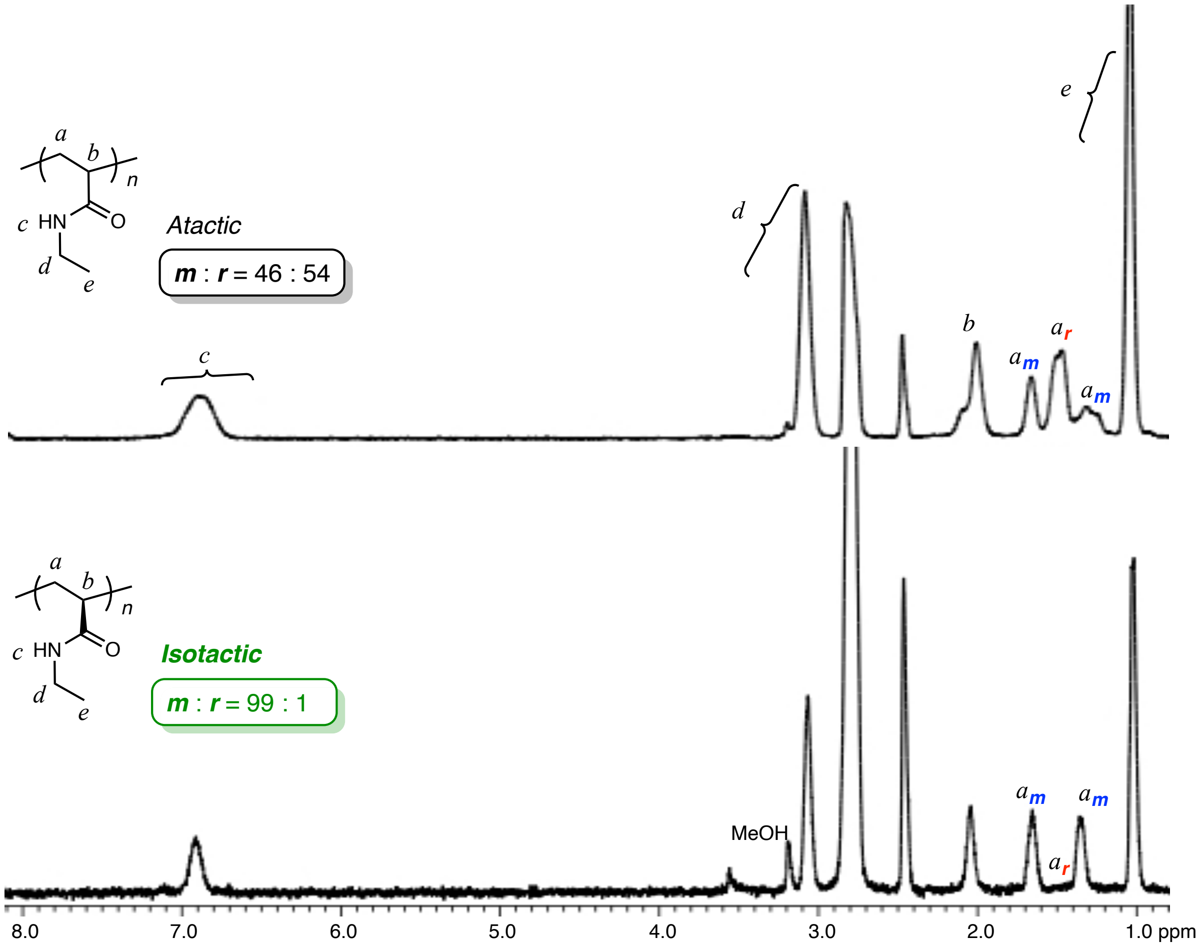

Figure S23. ^{1}H NMR spectrum (DMSO-d_6, 150 ˚C, lower) of the product via radical polymerization of **Ph**-BTDAm at –40˚C and subsequent aminolysis transformation with ethylamine in comparison with that of atactic poly(*N*-ethylacrylamide) (upper).

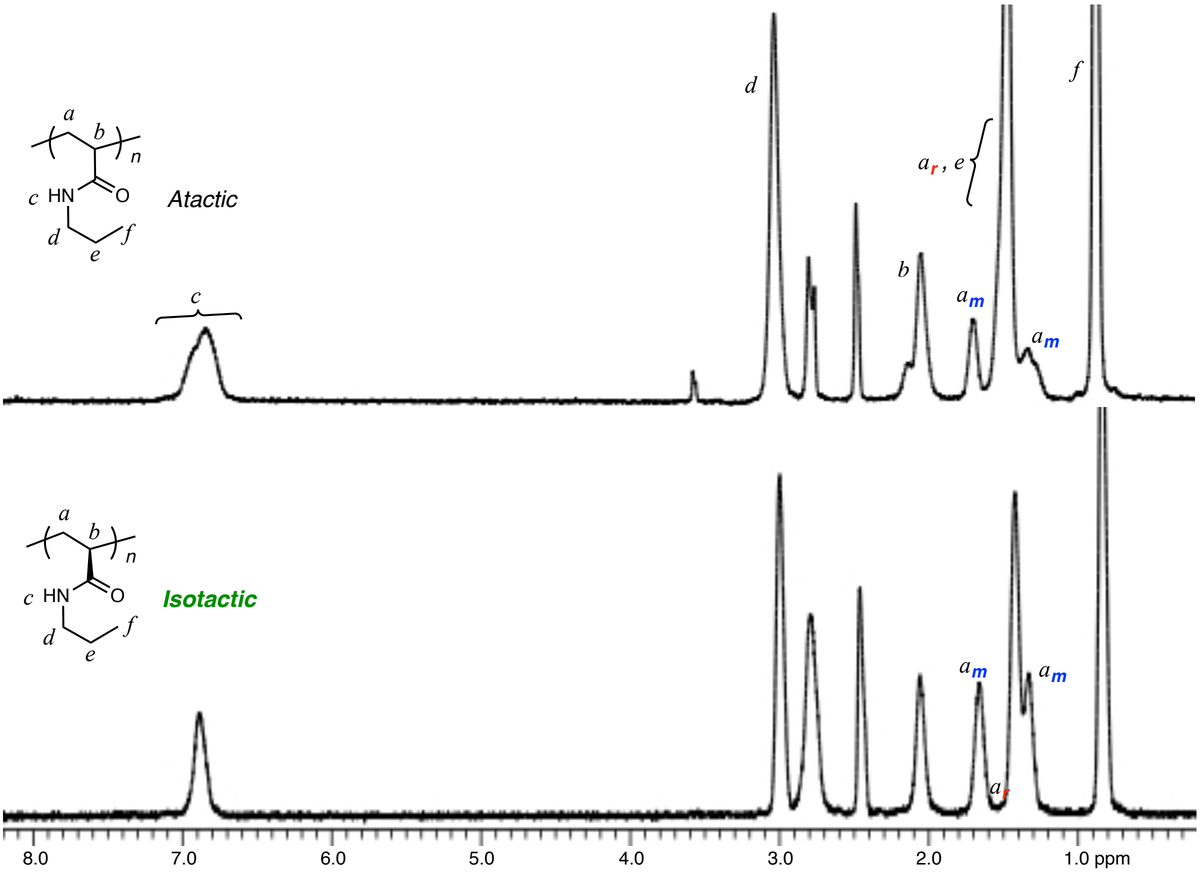

Figure S24. ^{1}H NMR spectrum (DMSO-d_6, 150 ˚C, lower) of the product via radical polymerization of **Ph**-BTDAm at –40˚C and subsequent aminolysis transformation with propylamine in comparison with that of atactic poly(*N*-propylacrylamide) (upper).

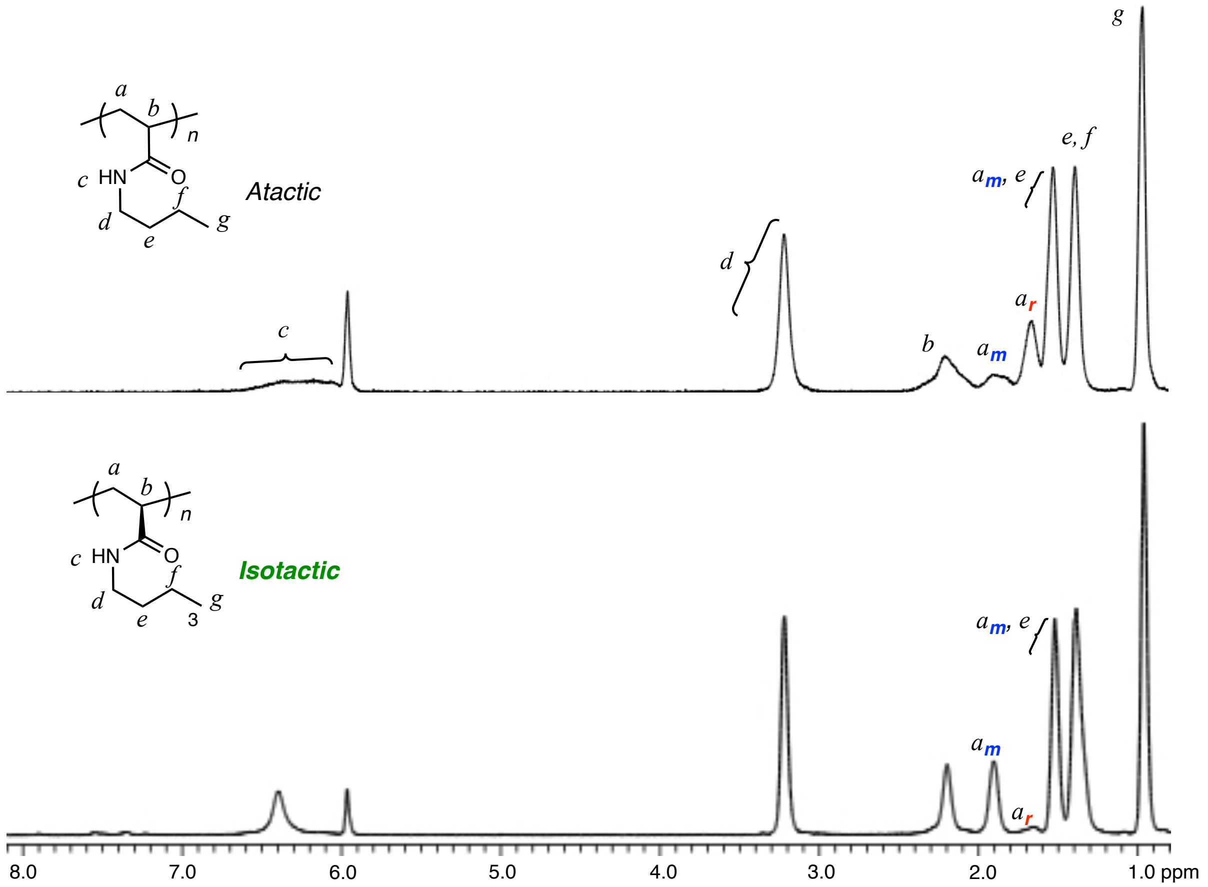

Figure S25. ^{1}H NMR spectrum ($(CDCl_2)_2$, 130 ˚C, lower) of the product via radical polymerization of **Ph**-BTDAm at –40˚C and subsequent aminolysis transformation with butylamine in comparison with that of atactic poly(*N*-butylacrylamide) (upper).

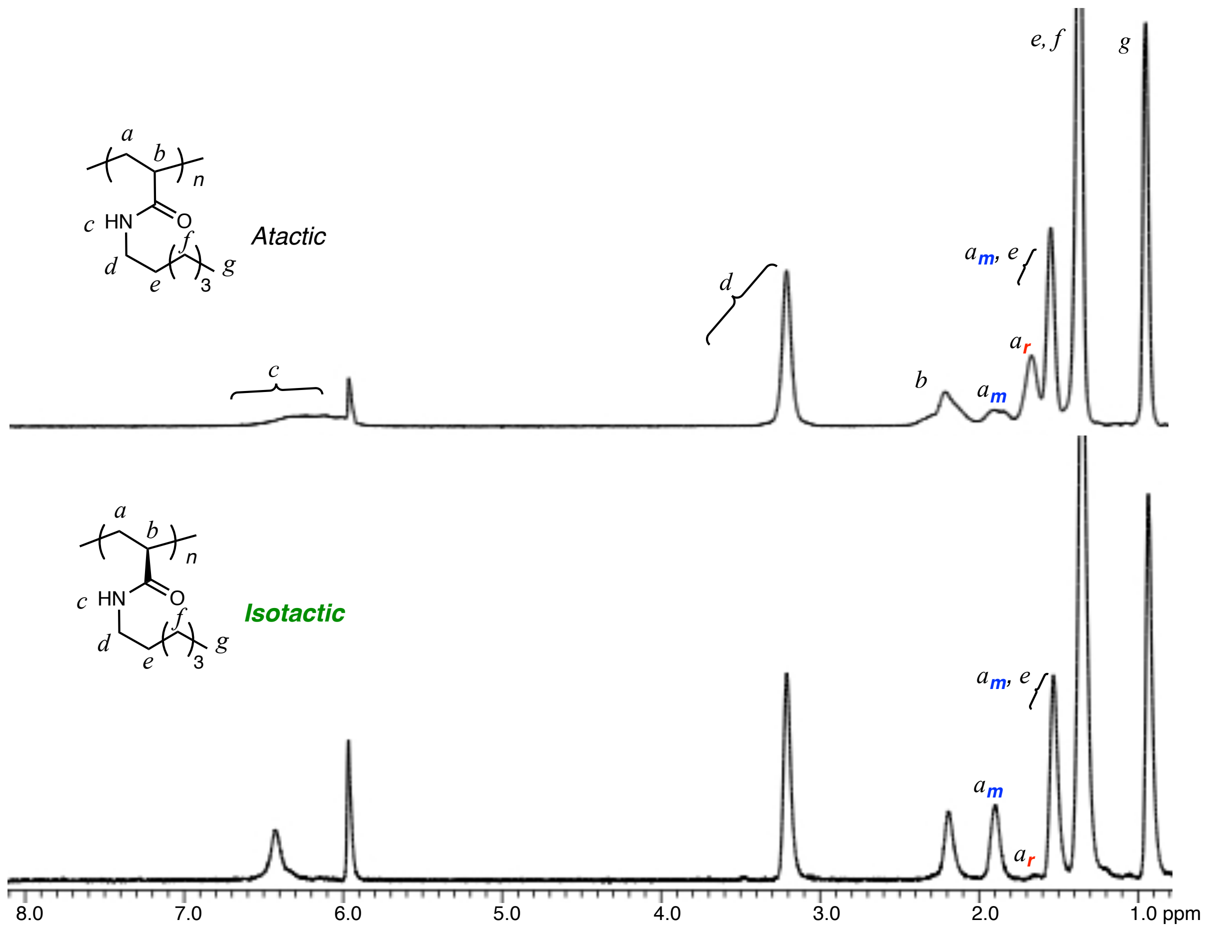

Figure S26. ^{1}H NMR spectrum ($(CDCl_2)_2$, 130 ˚C, lower) of the product via radical polymerization of **Ph**-BTDAm at –40˚C and subsequent aminolysis transformation with hexylamine in comparison with atactic poly(*N*-hexylacrylamide) (upper).

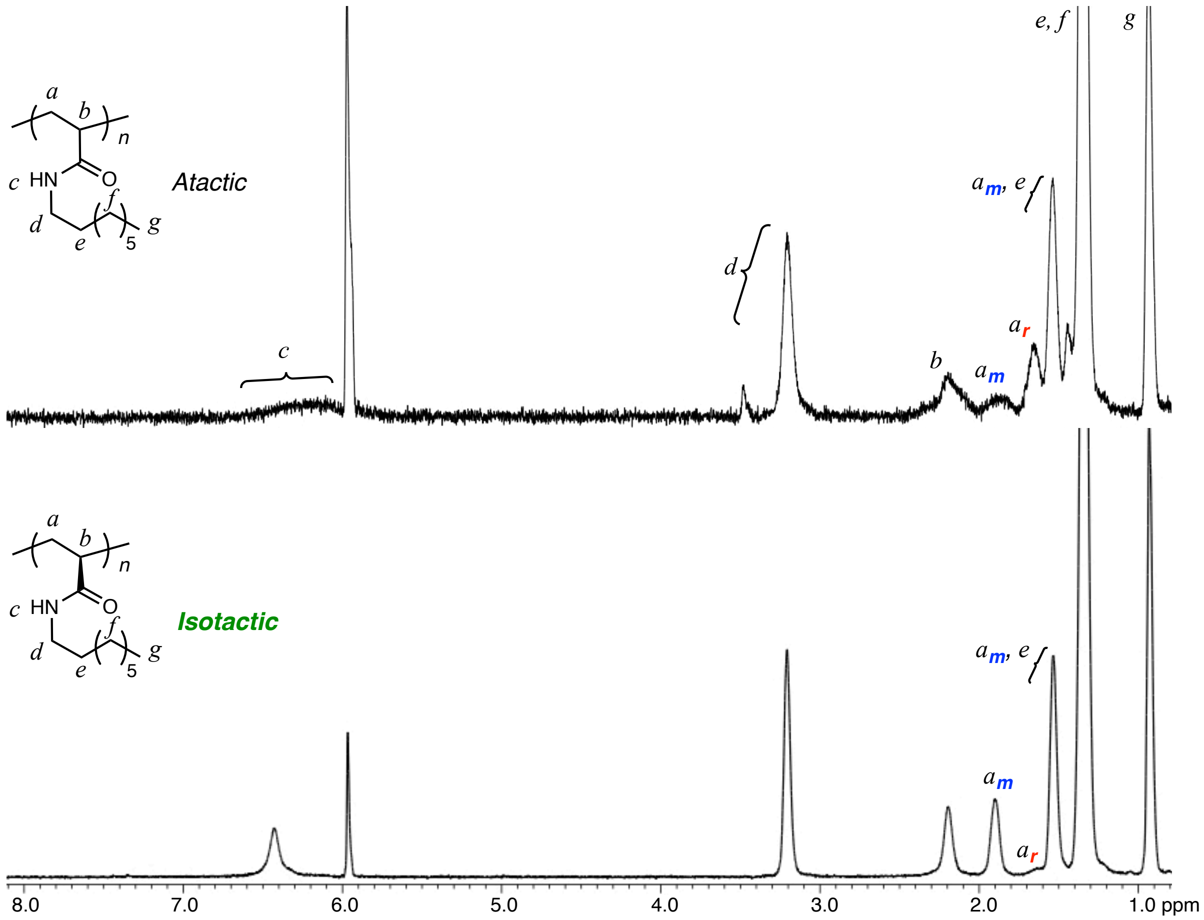

Figure S27. ^{1}H NMR spectrum ($(CDCl_2)_2$, 130 ˚C, lower) of the product via radical polymerization of **Ph**-BTDAm at –40˚C and subsequent aminolysis transformation with octylamine in comparison with that of atactic poly(*N*-octylacrylamide) (upper).

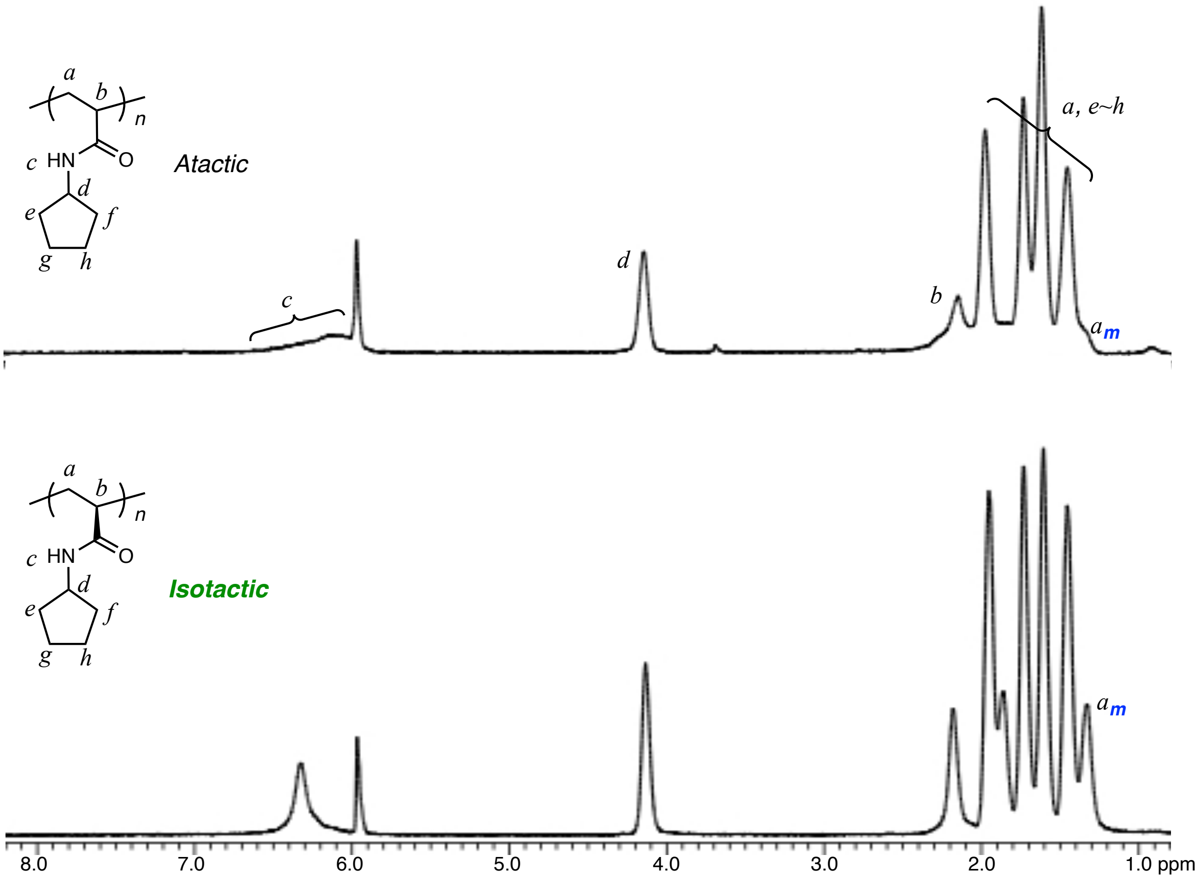

Figure S28. ^{1}H NMR spectrum ($(CDCl_2)_2$, 130 ˚C, lower) of the products via radical polymerization of **Ph**-BTDAm at –40˚C and subsequent aminolysis transformation with cyclopentylamine in comparison with that of atactic poly(*N*-cyclopentylacrylamide) (upper).

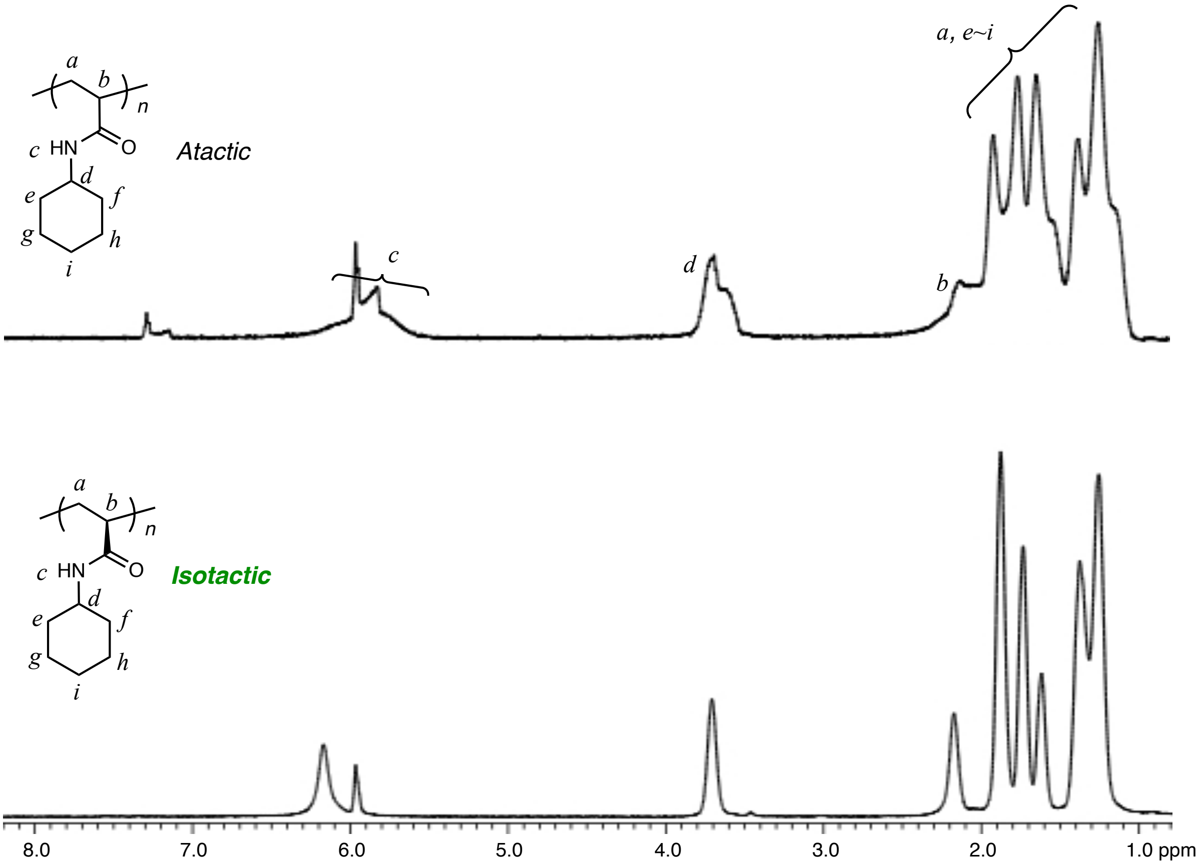

Figure S29. ^{1}H NMR spectrum ($(CDCl_2)_2$, 130 ˚C, lower) of the products via radical polymerization of **Ph**-BTDAm at –40˚C and subsequent aminolysis transformation with cyclohexylamine in comparison with that of atactic poly(*N*-cyclohexylacrylamide) (upper).

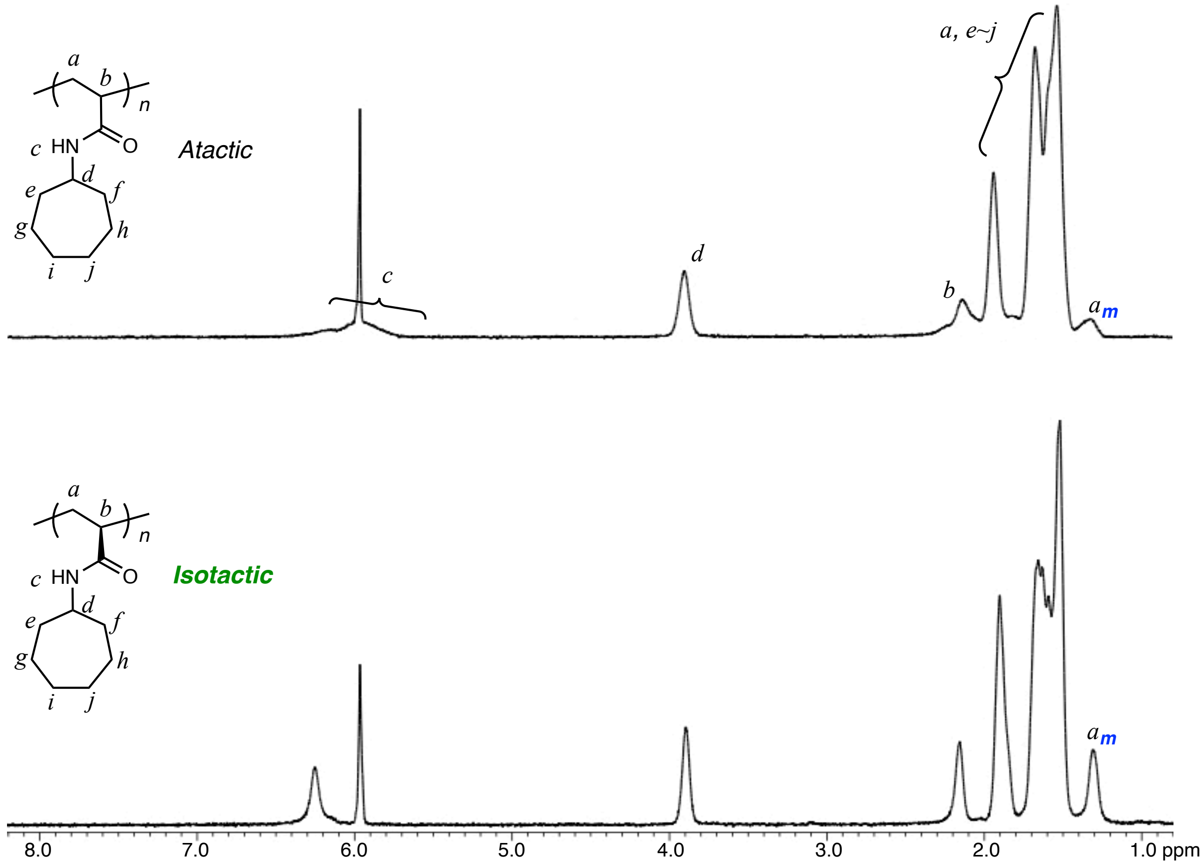

Figure S30. ^{1}H NMR spectrum ($(CDCl_2)_2$, 130 ˚C, lower) of the products via radical polymerization of **Ph**-BTDAm at –40˚C and subsequent aminolysis transformation with cycloheptylamine in comparison with that of atactic poly(*N*-cycloheptylacrylamide) (upper).

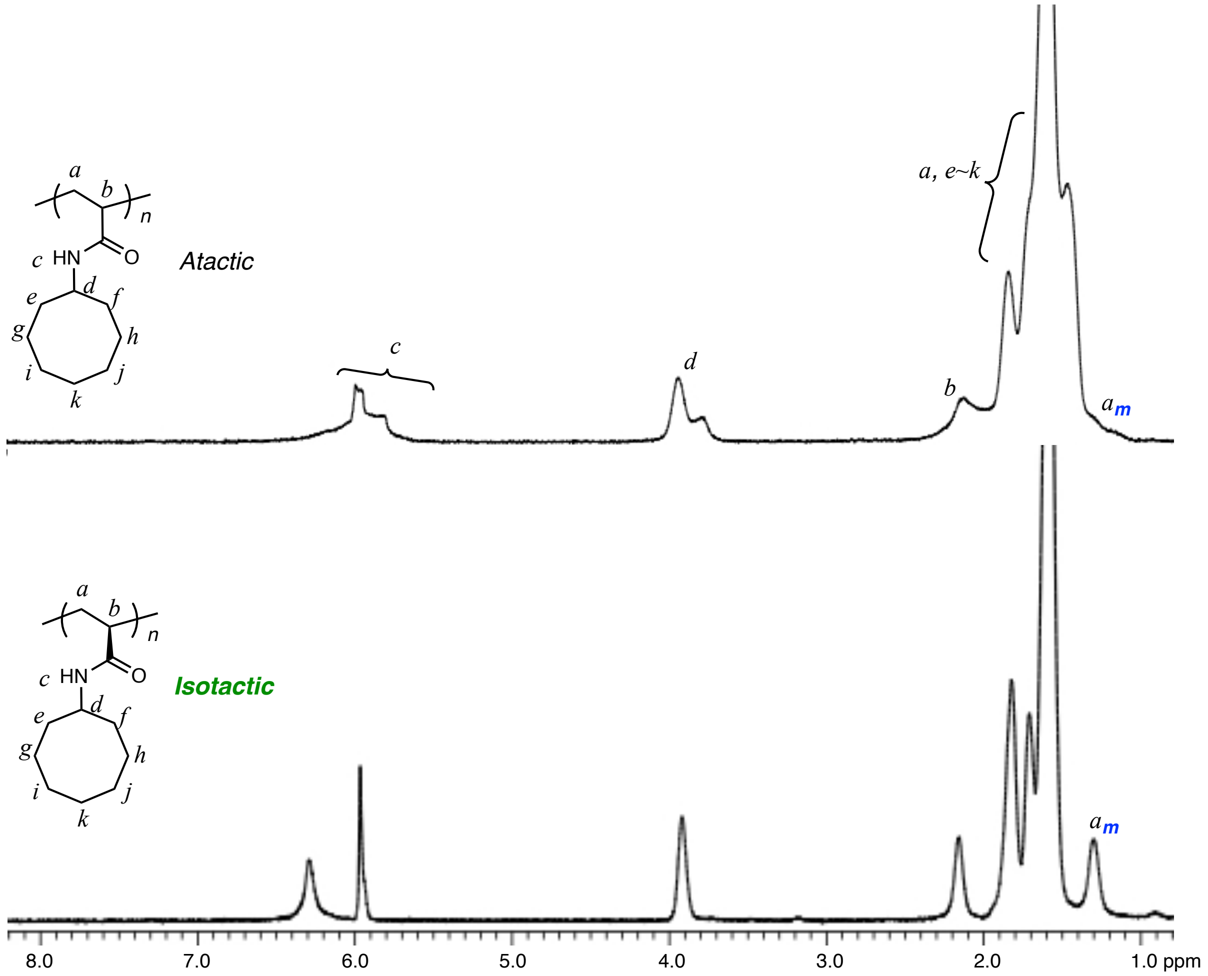

Figure S31. ^{1}H NMR spectrum ($(CDCl_2)_2$, 130 ˚C, lower) of the product via radical polymerization of **Ph**-BTDAm at –40˚C and subsequent aminolysis transformation with cyclooctylamine in comparison with that of atactic poly(*N*-cyclooctylacrylamide) (upper).

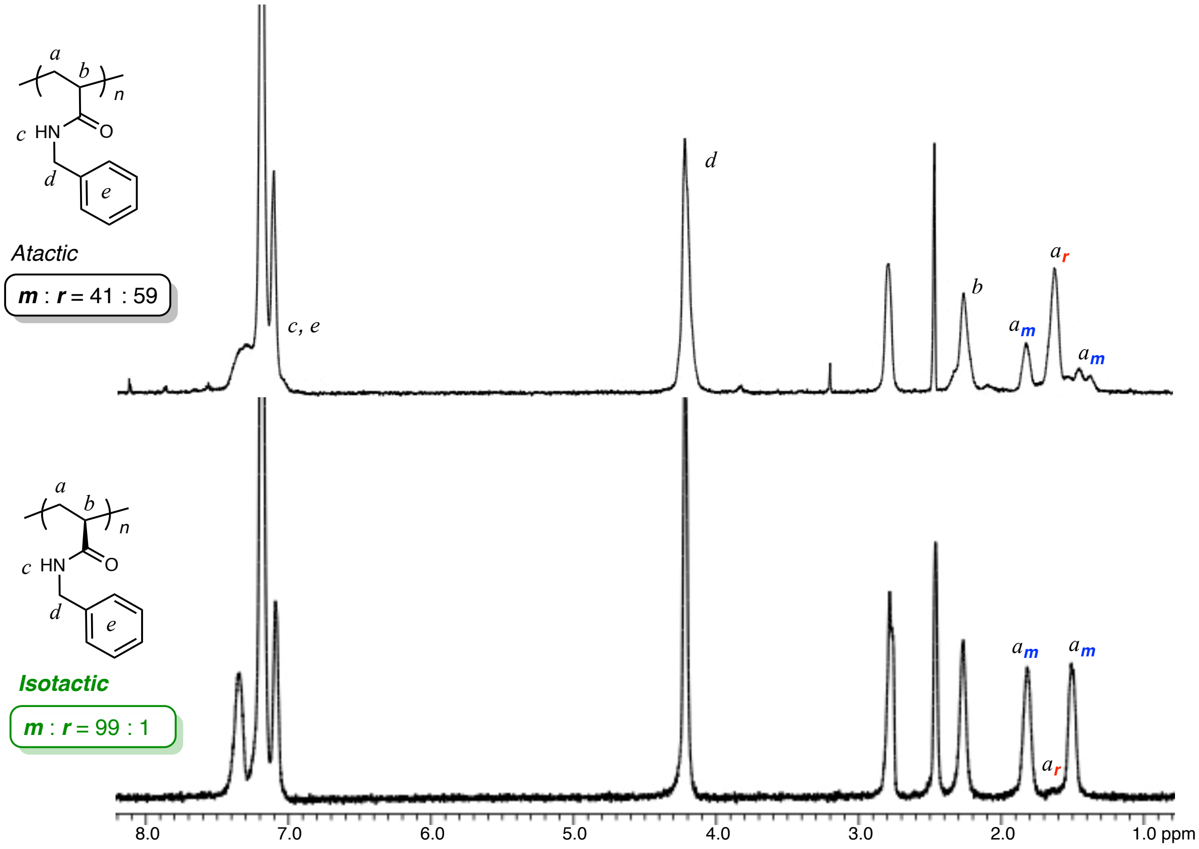

Figure S32. ^{1}H NMR spectrum (DMSO-d_6, 150 ˚C, lower) of the product via radical polymerization of **Ph**-BTDAm at –40˚C and subsequent aminolysis transformation with benzylamine in comparison with that of atactic poly(*N*-benzylacrylamide) (upper).

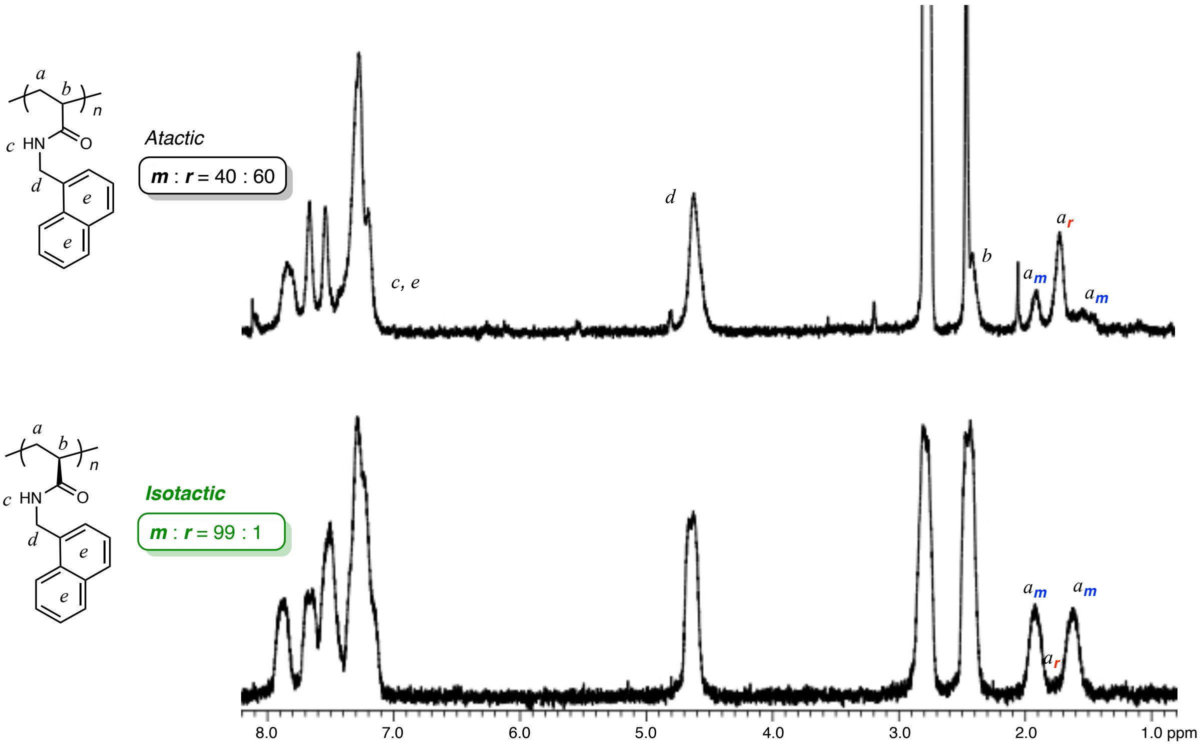

Figure S33. ^{1}H NMR spectrum (DMSO-d_6, 150 ˚C, lower) of the product via radical polymerization of **Ph**-BTDAm at –40˚C and subsequent aminolysis transformation with 1-naphthylamine in comparison with that of atactic poly(*N*-1-naphthylacrylamide) (upper).

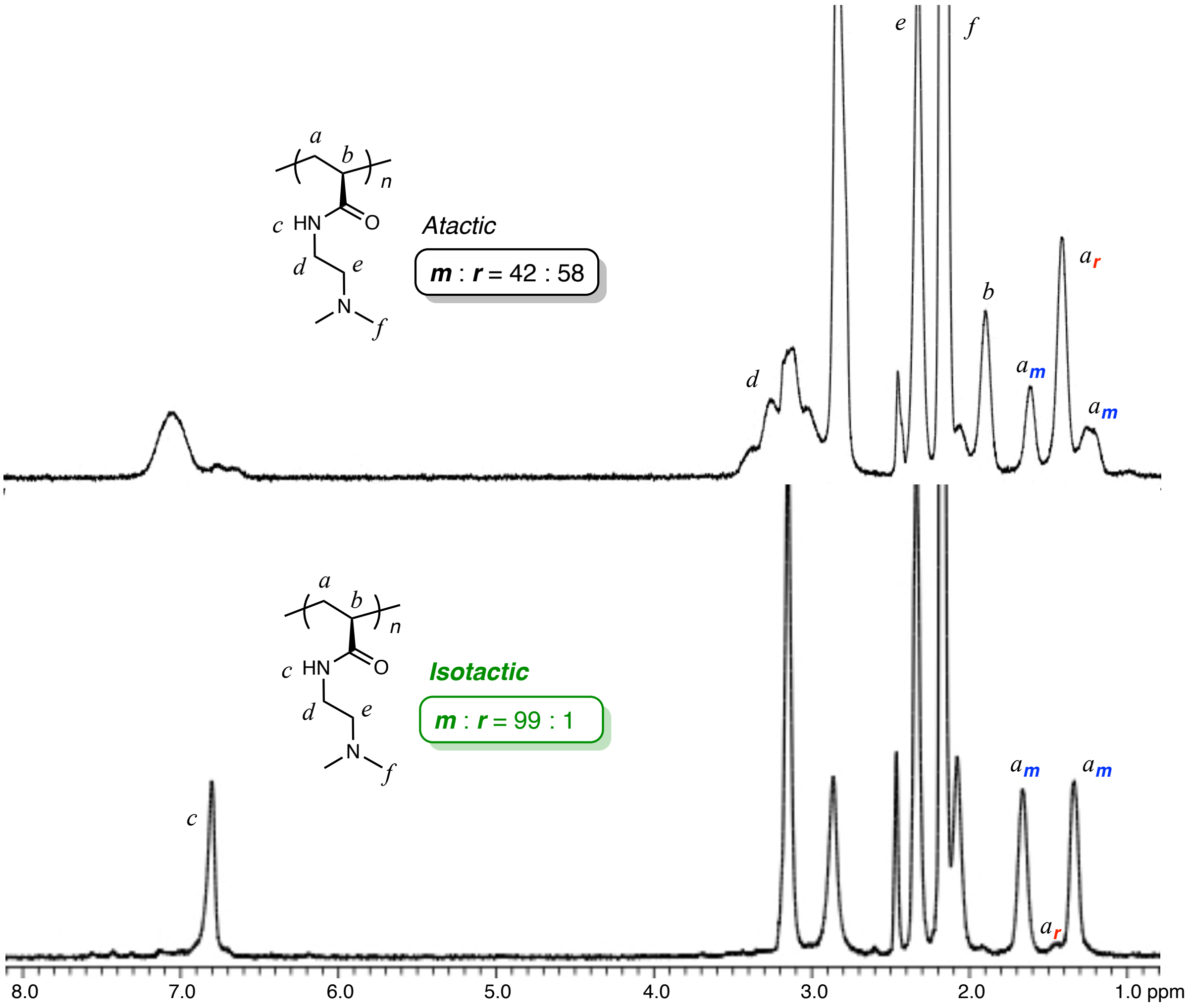

Figure S34. ^{1}H NMR spectrum (DMSO-d_6, 150 ˚C, lower) of the product via radical polymerization of **Ph**-BTDAm at –40˚C and subsequent aminolysis transformation with *N*,*N*-dimethylethylenediamine in comparison with that of atactic poly(*N*-[2-(dimethylamino)ethyl]acrylamide) (upper).

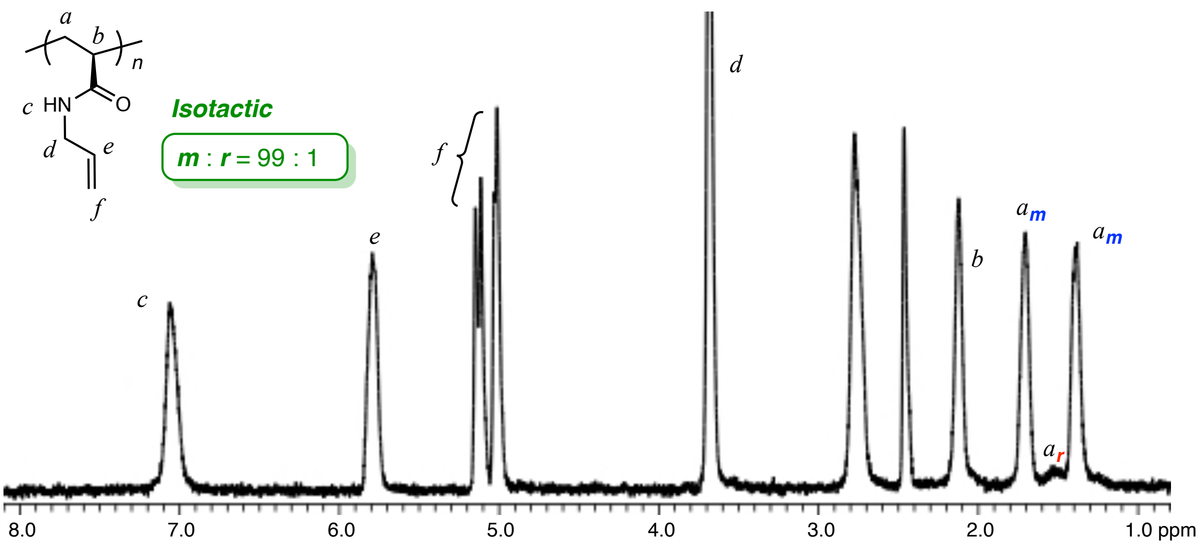

Figure S35. ^{1}H NMR spectrum (DMSO-d_6, 150 ˚C) of the product via radical polymerization of **Ph**-BTDAm at –40˚C and subsequent aminolysis transformation with allylamine.

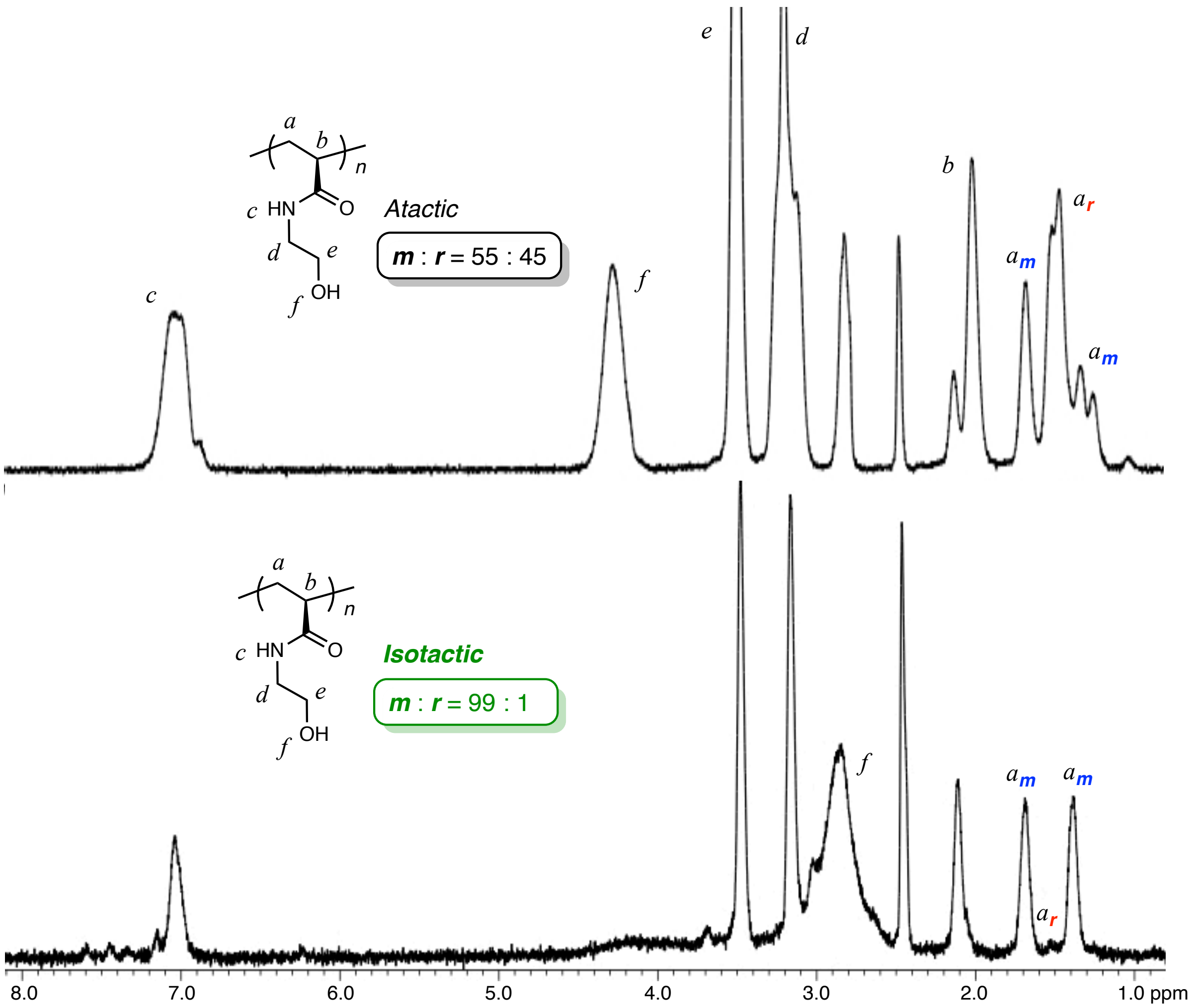

Figure S36. ^{1}H NMR spectrum (DMSO-d_6, 150 ˚C, lower) of the product via radical polymerization of **Ph**-BTDAm at –40˚C and subsequent aminolysis transformation with 2-aminoethanol in comparison with that of atactic poly[*N*-(2-hydroxyethyl)acrylamide) (upper).

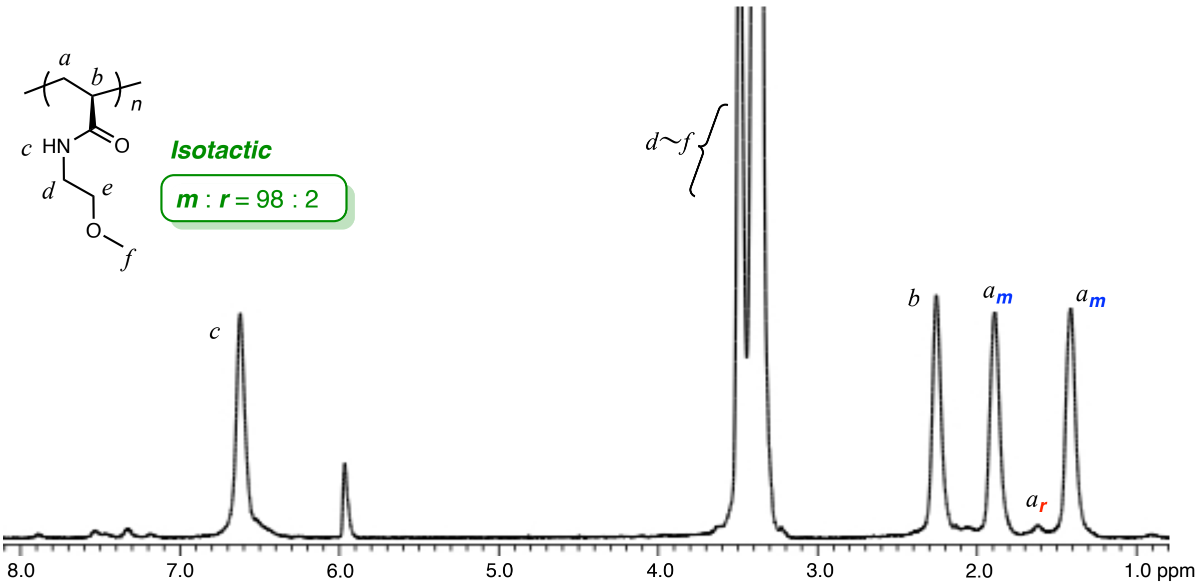

Figure S37. ^{1}H NMR spectrum (DMSO-d_6, 150 ˚C) of the product via radical polymerization of **Ph**-BTDAm at –40˚C and subsequent aminolysis transformation with 2-methoxyethylamine.

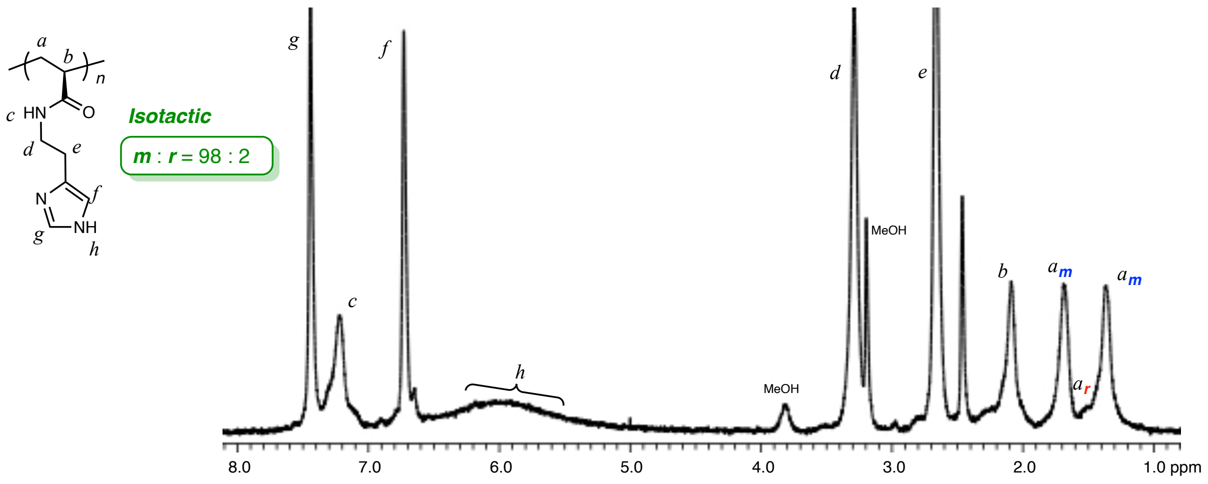

Figure S38. ^{1}H NMR spectrum (DMSO-d_6, 150 ˚C) of the product via radical polymerization of **Ph**-BTDAm at –40˚C and subsequent aminolysis transformation with histamine.

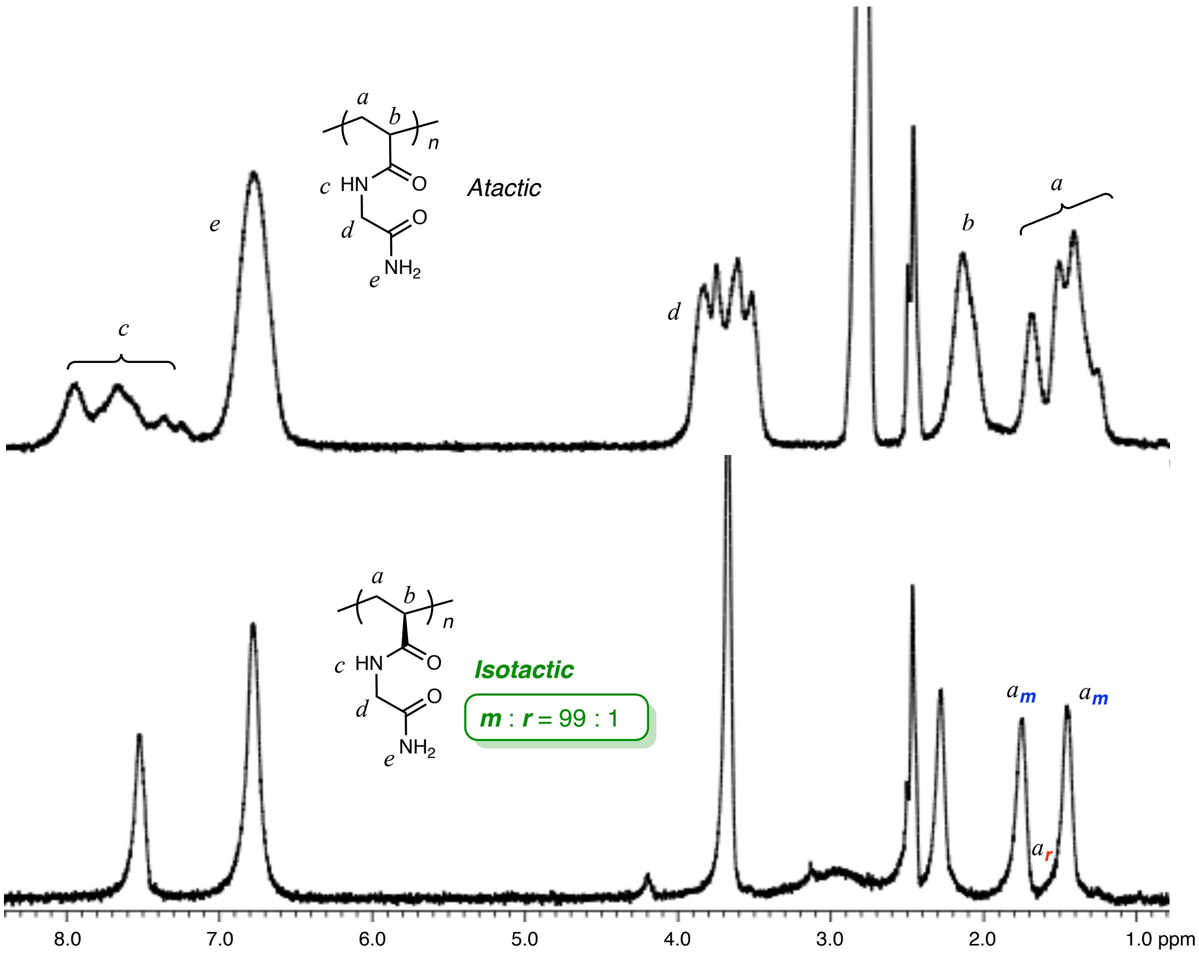

Figure S39. ^{1}H NMR spectrum (DMSO-d_6, 150 ˚C, lower) of the product via radical polymerization of **Ph**-BTDAm at –40˚C and subsequent aminolysis transformation with glycinamide hydrochloride and triethylamine in comparison with atactic poly(*N*-Acryloyl glycinamide) (upper).

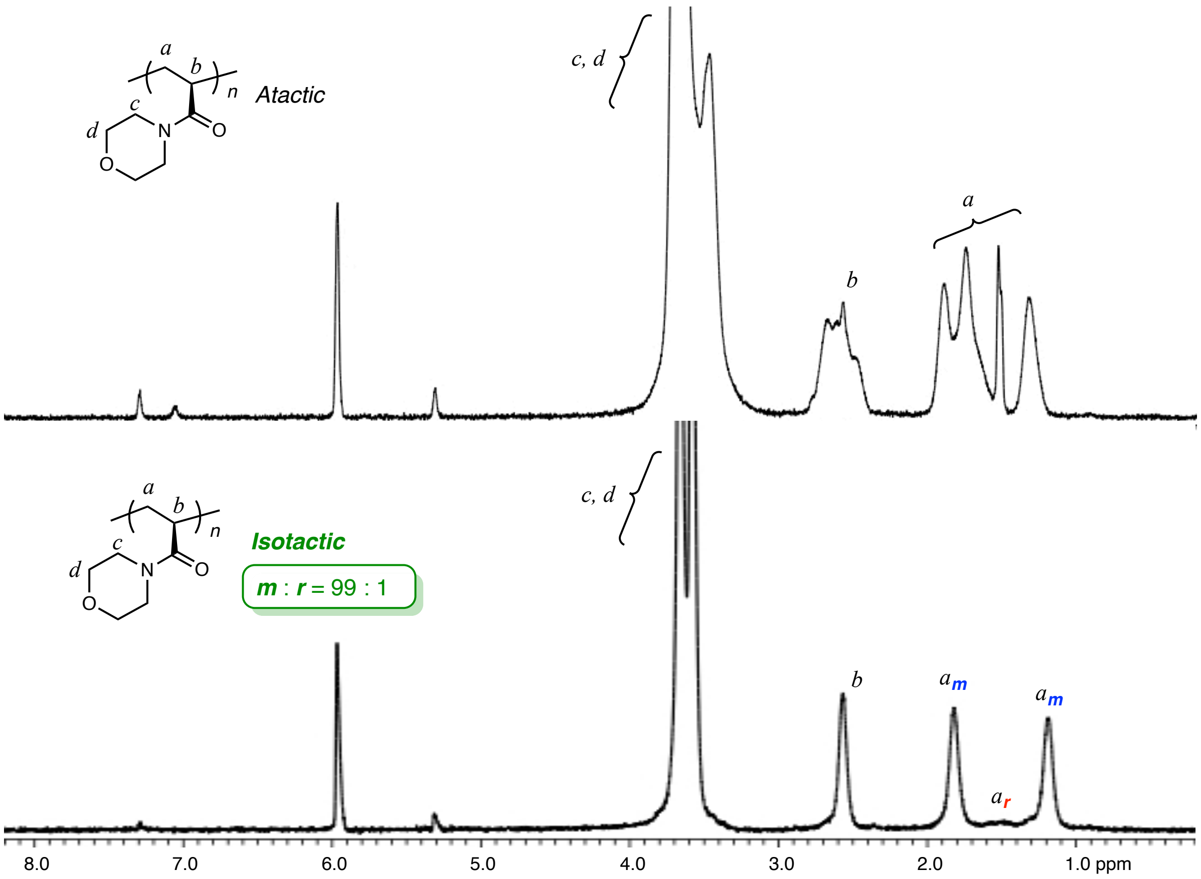

Figure S40. ^{1}H NMR spectrum ($(CDCl_2)_2$, 130 ˚C, lower) of the product via radical polymerization of **Ph**-BTDAm at –40˚C and subsequent aminolysis transformation with morpholine in comparison with atactic poly(4-acryloylmorpholine) (upper).

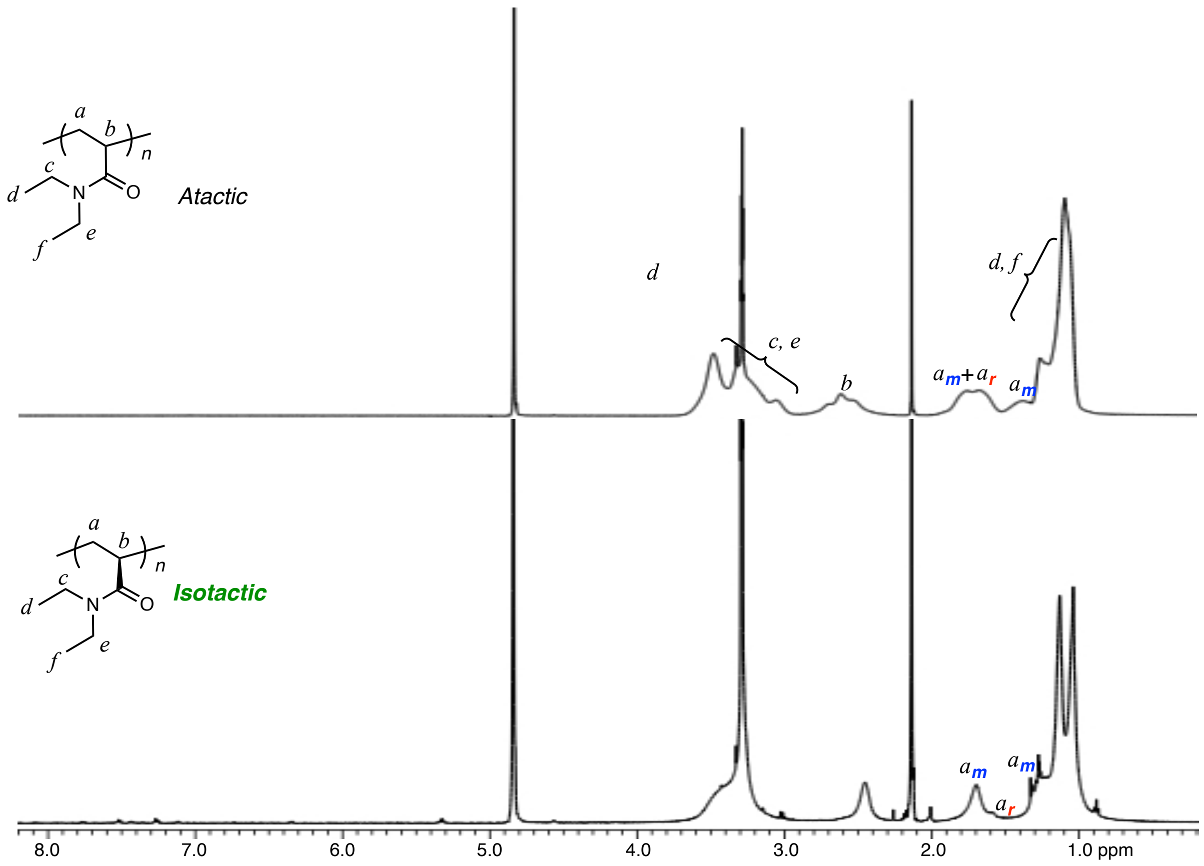

Figure S41. ^{1}H NMR spectrum (CD3OD, room temperature, lower) of the product via radical polymerization of **Ph**-BTDAm at –40˚C and subsequent aminolysis transformation with diethylamine in comparison with that of atactic poly(*N*,*N*-diethylacrylamide) (upper).

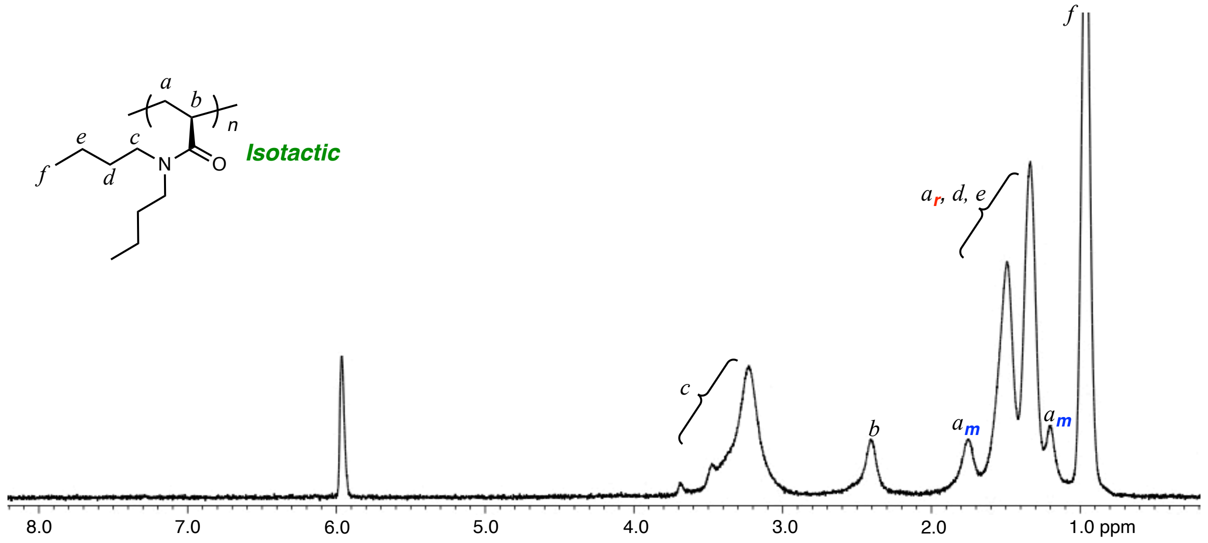

Figure S42. ^{1}H NMR spectrum ($(CDCl_2)_2$, 130 ˚C) of the product via radical polymerization of **Ph**-BTDAm at –40˚C and subsequent aminolysis transformation with dibutylamine.

References

1. Girard, Y., Atkinson, J. G. & Rokach, J. A new synthesis of 1,2,4-benzothiadiazines and a selective preparation of o-aminobenzenesulphonamides. *J. Chem. Soc., Perkin Trans. 1* 1043 (1979) doi:10.1039/p19790001043.

2. Pan, Y. & Ouchi, M. Stereospecific Radical Polymerization of a Side-Chain Transformable Bulky Acrylamide Monomer and Subsequent Post-Polymerization Modification for Syntheses of Isotactic Polyacrylate and Polyacrylamide. *Angewandte Chemie International Edition* **62**, e202308855 (2023).

3. Seuring, J., Bayer, F. M., Huber, K. & Agarwal, S. Upper Critical Solution Temperature of Poly(*N* -acryloyl glycinamide) in Water: A Concealed Property. *Macromolecules* **45**, 374–384 (2012).
